# Machine Learning for Risk Prediction in Colorectal Surgery—Game Changer or Unnecessary? An Institutional Pilot Study

**DOI:** 10.3390/diagnostics16193190

**Published:** 2026-10-01

**Authors:** He Ayu Xu, Philip Deslarzes, Jean Louis Raisaro, Dieter Hahnloser, Martin Hübner, Fabian Grass

**Affiliations:** 1 Biomedical Data Science Center, Lausanne University Hospital CHUV, University of Lausanne (UNIL), 1011 Lausanne, Switzerland; he.xu@chuv.ch (H.A.X.); jean.raisaro@chuv.ch (J.L.R.); 2 Department of Visceral Surgery, Lausanne University Hospital CHUV, University of Lausanne (UNIL), 1011 Lausanne, Switzerland; philip.deslarzes@chuv.ch (P.D.); dieter.hahnloser@chuv.ch (D.H.); martin.hubner@chuv.ch (M.H.)

**Keywords:** clusters, ERAS, trajectory, outcomes, colorectal surgery, recovery

## Abstract

**Background**: The aim of the present study was to identify machine learning (ML)-driven risk constellations to predict specific postoperative outcomes and recovery targets to support the development of targeted interventions, optimized to individual risk profiles. **Methods**: This retrospective institutional cohort study included consecutive patients who underwent elective colorectal surgery over an 11-year study period focusing on baseline patient characteristics, intraoperative details, and postoperative complications. Five supervised classification algorithms for specific complications and recovery targets were used: logistic regression, support vector machine (SVM), XGBoost, decision tree, and gradient boosting. The dataset was randomly divided into training (80%) and testing (20%) subsets. Model performance was assessed using F1-score for classification task, and for each prediction target; the best-performing model and hyperparameter combination were selected based on validation results. To interpret the contribution of individual features to model predictions, SHAP (Shapley Additive exPlanations) analysis was applied to the best-performing model identified for each prediction target. **Results**: Logistic regression was the best-performing model for six of the seven postoperative outcomes, whereas SVM performed best for postoperative ileus, indicating that optimal model selection was outcome-specific. Overall, however, none of the tested models clearly outperformed the others in their predictive potential. The discriminative power of the models, as measured by ROC-AUC (receiver operating characteristics–area under the curve), was consistently higher than the baseline of 0.50, peaking at 0.93 for renal dysfunction. In terms of classification accuracy, the prediction of postoperative ileus was the most robust, yielding an F1-score of 0.87. For mobilization, the model showed balanced performance across F1-score (0.72), precision (0.74), and recall (0.71). Given the limited performance across all selected models, SHAP analysis was used to improve the interpretability of the predictions to better understand the contributions of each clinical variable. **Conclusions**: Complex models in the setting of a structured perioperative dataset only slightly outperform simpler approaches, highlighting the importance of data quality and feature richness rather than just increased model complexity. Future work on improving ML-based risk prediction strategies should thus ensure data quality and focus on integrating dynamic perioperative and postoperative measurements, exploring more flexible modeling frameworks, and validating model performance in larger external cohorts.

## 1. Introduction

Risk prediction in surgery is mandatory to anticipate challenges, launch timely preventive measures, mitigate modifiable risk factors and tailor perioperative care [1]. While conventional risk prediction tools are mainly driven by clinical experience based on intuitive linear risk constellations, machine learning (ML)-based artificial intelligence (AI) tools empower refined data processing and risk prediction without human input. A systematic review of ML applications in colorectal surgery revealed various algorithms incorporating neural networks, random forests, and gradient boosting methods to predict postoperative complications, disease staging, and treatment responses [2].

Our group used unsupervised ML strategies to identify patient clusters with distinct demographic profiles and compliance patterns in an enhanced recovery pathway, which were strongly associated with functional and clinical outcomes [3]. This analysis represented a first step to personalized and tailored perioperative care pathways, which may be particularly beneficial for intermediate- and high-risk patients. Through improved performance and precision, ML-based risk prediction strategies may help to decrease postoperative morbidity further. Ideally, such ML-driven models ultimately extract data from electronic medical records to dynamically improve risk prediction throughout the surgical journey. Hence, our group aimed to identify ML-driven risk constellations to predict specific postoperative outcomes and recovery targets with an intent to further support the development of targeted interventions, optimized to individual risk profiles.

## 2. Methods

This retrospective institutional cohort study represents an institutional quality improvement initiative including consecutive patients who underwent elective colorectal surgery at the Department of Visceral Surgery, Lausanne University Hospital (CHUV), over an 11-year study period (1 May 2011–31 December 2022).

The institutional Enhanced Recovery After Surgery (ERAS) protocol was implemented and established as perioperative standard of care since the beginning of the study period [4]. The ERAS protocol was developed in accordance with the ERAS Society’s published guidelines [5]. Since implementation, data from all colorectal surgical patients have been prospectively recorded in a dedicated online database (ERAS Interactive Audit System, EIAS), with periodic data auditing every 2 to 4 weeks to check for data accuracy and completeness. Data collection was performed by a dedicated ERAS coordinator, who systematically recorded baseline patient characteristics, intraoperative details, and postoperative complications In brief, postoperative ileus (POI) was defined as nasogastric tube reinsertion [6], complications were graded according to the Clavien/Dindo classification into overall (grade I–V) and major (≥IIIb) complications [7], while respiratory [8] and renal complications [9] were defined according to previous institutional publications. Only patients who provided general research consent were included. This study was approved by the local ethics committee (CER-VD 2024-01803). All data points were extracted from EIAS using standardized definitions across centers [10].

### 2.1. Statistical Analysis

#### 2.1.1. Data Preprocessing, Model Selection and Hyperparameter Tuning

All models were built and tested with python 3.8.19, scikit-learn package version 1.4.2 and shap package version 0.44.1.

Prior to model development, the dataset was preprocessed to ensure data quality and consistency. All rows containing missing or empty values were removed from the dataset for each prediction target and model. This step ensured that all models were trained and evaluated on complete cases, avoiding potential biases or errors introduced by imputed data. For each prediction target, we selected different predictors based on medical and clinical considerations; the detailed list is presented in Table 1.

We evaluated five supervised machine learning classification models: logistic regression, support vector machine (SVM), XGBoost, decision tree, and gradient boosting. In this study, supervised classification refers to models trained using patient characteristics together with known postoperative outcomes, allowing the models to learn patterns associated with each outcome.

Logistic regression was included as a candidate algorithm within the machine learning framework and was subjected to the same preprocessing, model training, hyperparameter optimization, cross-validation, and evaluation procedures as the other algorithms. Although logistic regression is a traditional statistical prediction method, it was treated in this study as a comparatively simple and interpretable machine learning model.

The dataset was randomly divided into a training set (80%) for model development and a testing set (20%) for performance evaluation for each prediction target.

To assess potential collinearity and redundancy among predictors, we performed additional dependency analyses before final model development. For numerical predictors, person correlation analysis was conducted, and variance inflation factors (VIFs) were calculated after appropriate preprocessing of continuous variables. For categorical predictors, pairwise associations were assessed using Cramér’s V. Strong collinearity or dependency was predefined as an absolute correlation coefficient > 0.8, VIF > 10, or Cramér’s V > 0.5. Predictors showing evidence of substantial redundancy were planned to be reviewed based on clinical relevance, missingness, and interpretability before model inclusion.

Since all the prediction targets were binary (meaning the values are either 0 or 1, where 0 means ‘no’ and 1 means ‘yes’), we implemented the binary classifiers in the following steps. First, we used one-hot encoding to encode the categorical variables (such as ‘smoking’, ‘peritoneal soiling/contamination’ and ‘mobilization on POD2′), then we standardized the numerical variables (such as ‘age’, ‘weight change on POD2′, ‘oral fluids taken on POD2′, etc.) by removing the mean and scaling to unit variance.

**Figure 1:**
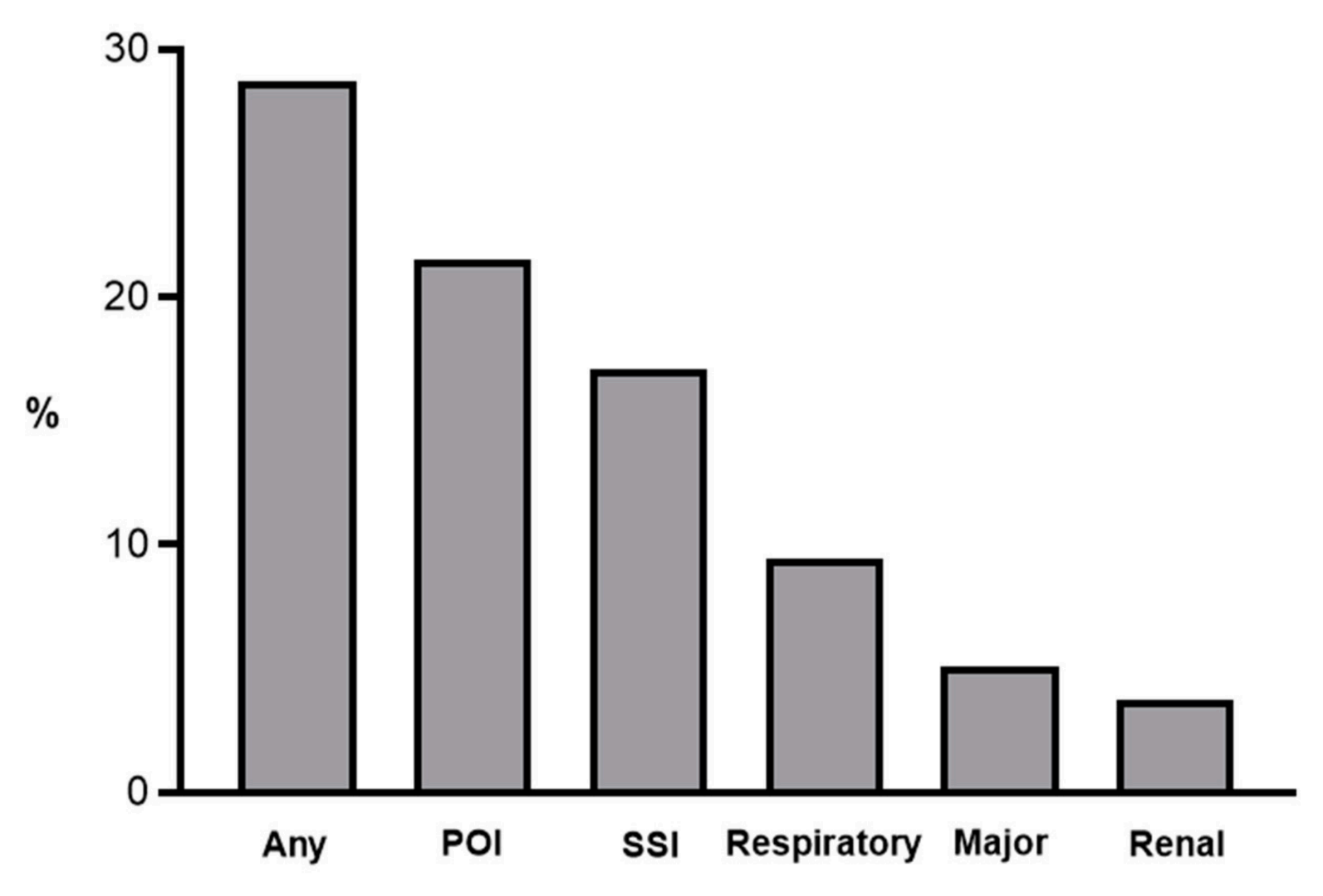
Postoperative complications of the entire cohort (%). POI—postoperative ileus; any—Clavien I–V; SSI—surgical site infection; major—Clavien IIIb-V.

To address class imbalance among the prediction targets, we evaluated three strategies: (1) no resampling, (2) cost-sensitive learning using the scikit-learn built-in parameter class_weight = “balanced”, and (3) the Synthetic Minority Over-sampling Technique (SMOTE) [9]. As several outcomes exhibited substantial class imbalance (Figure 1), the performance of these approaches was systematically compared for each prediction target (Supplementary Material). The comparison demonstrated that models using class_weight = “balanced” consistently achieved comparable or superior performance relative to the other approaches, whereas SMOTE did not provide additional predictive benefit. Therefore, the final analyses reported in this study were conducted using the class_weight = “balanced” approach [11]. Classifier models were developed using model-specific predefined hyperparameter search grids, including regularization strength and solver for logistic regression; tree depth and minimum split size for decision trees; regularization strength and kernel type for support vector machines; and number of estimators, learning rate, and maximum tree depth for gradient boosting and XGBoost. Model training and hyperparameter tuning were performed using repeated stratified five-fold cross-validation (RepeatedStratifiedKFold in scikit-learn), which preserved the class distribution within each fold. A five-fold strategy was chosen rather than a larger number of folds because several prediction targets were highly imbalanced, and this approach helped ensure that each fold contained an adequate number of positive cases. The details of the hyperparameter search grids are presented in the Supplementary Material.

We also implemented two baseline models to provide baseline comparisons. The first baseline model is a model that makes random guesses, and the second baseline model is a model that always predicts the majority class.

Model performance was evaluated using precision, recall (sensitivity), specificity, F1-score, and the area under the receiver operating characteristic curve (ROC-AUC). For each prediction target, the model and hyperparameter combination achieving the best performance on the validation set were selected for subsequent analyses. Because classification metrics such as precision, recall, specificity, and F1-score are threshold-dependent, model-specific decision thresholds were optimized using the validation data. This approach avoided overly sensitive operating points that could lead to clinically unrealistic prediction behavior, particularly for rare outcomes.

#### 2.1.2. Model Interpretation with SHAP Analysis

To interpret the contribution of individual features to model predictions, we applied SHAP (Shapley Additive exPlanations) analysis to the best-performing model identified for each prediction target [12,13]. We prioritized SHAP over standard correlation analysis because SHAP accounts for the multi-dimensional nature of the model, capturing non-linear relationships and interaction effects. Correlation is limited to assessing isolated linear associations; in contrast, SHAP identifies the importance of a feature in the presence of others, providing a more accurate representation of feature importance in high-dimensional predictive modeling. To increase usability in clinical practice, a graphical representation of prediction targets of SHAP feature importance was created.

## 3. Results

In total, 1732 consecutive patients underwent elective colorectal surgery over the study period. After applying exclusion criteria (no research consent), 1381 patients were available for final analysis. Table 2 displays the proportions of missing data and the distributions for the predictor variables:

Figure 1 displays the target variables. The POD 2 mobilization target of ≥6 h (not displayed) was achieved by 60.2% of patients.

The collinearity assessment did not identify strong redundancy among the included predictors. No numerical predictor pairs showed strong correlation, and VIF values did not indicate substantial multicollinearity. Similarly, Cramér’s V analysis did not reveal strong dependency among categorical predictors (see Supplementary Materials). Therefore, all clinically relevant predictors were retained in the final model development.

### 3.1. Predictive Models

We compared the performance of all models with different parameter combinations, and selected the best-performing model based on the F1-score. The best-performing model for each target prediction is presented in Table 3. In general, the logistic regression model outperformed the other models such as boosting-based models and SVM for most of the prediction cases.

Predictive performance varied across postoperative outcomes. The mean ROC-AUC exceeded the random-guess value of 0.5 for all prediction targets. The highest ROC-AUC values were observed for major complications and any complication, at 0.891 ± 0.063 and 0.844 ± 0.043, respectively. For renal dysfunction, the ROC-AUC was 0.807 ± 0.075. However, the F1-score for this outcome was lower than its ROC-AUC might suggest, which may be related to the low event rate and resulting class imbalance.

For surgical site infection (SSI), the model achieved a ROC-AUC of 0.787 ± 0.051, with a recall of 0.697 ± 0.085 and specificity of 0.811 ± 0.089, suggesting a relatively balanced ability to identify both positive and negative cases. The model for postoperative ileus (POI) reached an F1-score of 0.626 ± 0.116 and a ROC-AUC of 0.782 ± 0.100, with specificity 0.921 ± 0.059 and moderate recall 0.627 ± 0.174.

Performance was more limited for respiratory complications, with a ROC-AUC of 0.743 ± 0.084 and F1-score of 0.441 ± 0.125. For mobilization on postoperative day 2, the model achieved the highest F1-score (0.820 ± 0.022) and high recall (0.978 ± 0.039), but specificity was low (0.192 ± 0.198), indicating that the model tended to classify most patients as positive for this outcome. Therefore, although sensitivity was high, the model had limited ability to correctly identify negative cases.

Overall, these results suggest that the developed models demonstrated meaningful predictive performance for several postoperative outcomes, particularly major complications, any complication, renal dysfunction, SSI, and POI. However, performance differed substantially by outcome, and for some targets, especially mobilization on postoperative day 2, the imbalance between sensitivity and specificity should be considered when interpreting potential clinical utility.

### 3.2. SHAP Analysis

Feature importance for predicting any complication showed that SHAP analysis identified absence of peritoneal soiling/contamination, oral fluid intake on POD2, and minimally invasive surgical approach as the strongest contributors to lower predicted risk. In contrast, presence of peritoneal soiling/contamination, greater weight change on POD2, ASA class 3, open surgical approach, and ASA class 4 were associated with higher predicted risk, although the latter features had smaller impact strengths. These results indicate that contamination status, surgical approach, oral intake, and postoperative recovery markers contributed substantially to the model output.

Regarding feature importance for predicting major complications, peritoneal contamination status was one of the strongest contributors to model predictions. Absence of peritoneal soiling/contamination was associated with lower predicted risk, whereas presence of contamination was associated with higher predicted risk. Other important contributors included ASA physical status, surgical approach (open vs. minimally invasive surgery (MIS), surgical setting (elective vs. emergency), and early postoperative recovery variables. Minimally invasive surgery, mobilization on POD2, and higher oral fluid intake on POD2 were generally associated with lower predicted risk, while greater postoperative weight change was associated with higher predicted risk. These results indicate that both operative factors and early postoperative recovery indicators contributed meaningfully to the model output.

For predicting respiratory complications, mobilization on POD2 was the strongest contributor to model predictions, followed by smoking-related variables, oral fluid intake on POD2, age, and weight change on POD2. Mobilization on POD2, no smoking status, and greater oral fluid intake on POD2 were associated with lower predicted risk, whereas older age and greater postoperative weight change were associated with higher predicted risk. The effects of smoking and no mobilization variables were smaller in magnitude. These results indicate that postoperative recovery markers, particularly mobilization and oral intake, together with patient-level factors such as age and smoking status, contributed meaningfully to the model predictions.

To predict the status of mobilization on POD2, oral fluid intake on POD2 and total intravenous fluid volume on postoperative day 0 had the greatest impact on model predictions. Higher oral fluid intake on POD2 was associated with higher predicted risk, whereas greater intravenous fluid volume on day 0 was associated with lower predicted risk. Age, weight change on POD2, preoperative weight change, and BMI showed smaller but relevant contributions. Older age and higher BMI were associated with increased predicted risk, while greater postoperative and preoperative weight change were associated with lower predicted risk. These results indicate that fluid-related perioperative variables, together with selected patient-level characteristics, contributed substantially to the model output.

To predict POI, SHAP-based feature importance analysis showed that oral fluid intake on POD2 had the greatest influence on model output, followed by total intravenous fluid volume on postoperative day 0, BMI, age, and weight change on POD2. Higher oral fluid intake on POD2, BMI, and age were associated with lower predicted risk, whereas higher intravenous fluid volume on day 0 and greater weight change on POD2 were associated with higher predicted risk. Mobilization-related variables on POD2 showed comparatively small contributions to the model predictions.

To predict renal dysfunction, total intravenous fluid volume on POD0 had the greatest impact on model predictions, followed by weight change on postoperative day 2, mobilization status on POD2, and oral fluid intake on POD2. Higher intravenous fluid volume and greater weight increase by POD2 were associated with higher predicted risk, whereas oral fluid intake and mobilization on POD2 were associated with lower predicted risk. Conversely, absence of mobilization on POD2 was associated with higher predicted risk. These findings indicate that variables reflecting early postoperative fluid status and functional recovery were among the main drivers of the model predictions.

To predict SSI, length of incision had the greatest impact on model predictions, with greater incision length associated with higher predicted risk. Mobilization-related variables on POD2, oral fluid intake on POD2, and weight change on POD2 had smaller but relevant contributions. Mobilization and greater oral intake on POD2 were associated with lower predicted risk, whereas absence of mobilization and greater postoperative weight change were associated with higher predicted risk. These results indicate that surgical factors and early postoperative recovery indicators contributed substantially to the model output.

**Figure 2:**
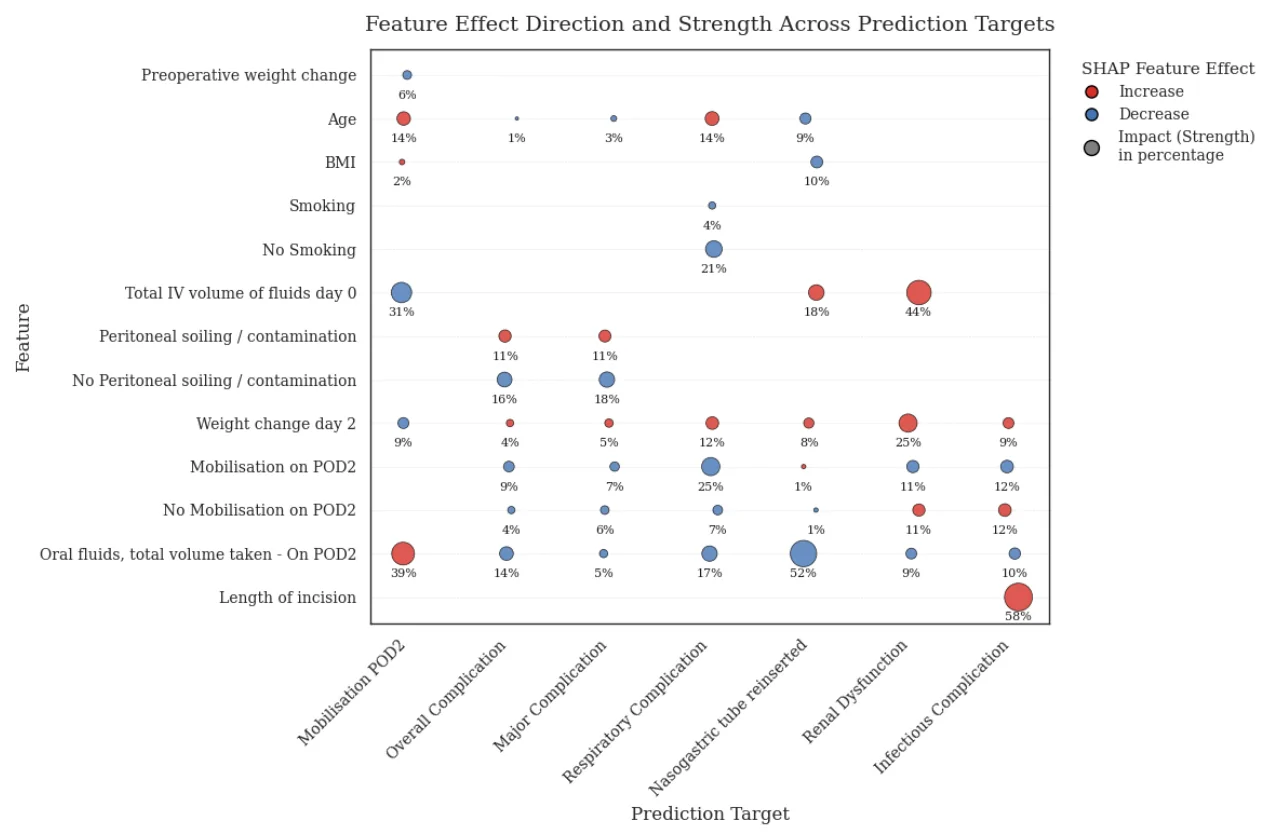
Summary of SHAP feature importance across multiple prediction targets. Bubble size represents the relative impact strength (percentage contribution) of each feature, while color indicates the direction of effect on the model prediction (red: increase, blue: decrease). The figure highlights both shared and outcome-specific predictors, illustrating how clinical features differentially influence any, major, respiratory and infectious complications, delayed mobilization, and renal dysfunction. BMI—body mass index; POD—postoperative day.

All results are summarized in Figure 2. To optimize the clarity and interpretability of the main figure, we presented a selected subset of predictors that were consistently used across most prediction targets. Variables such as diabetes status, ASA physical status, surgical approach, and surgical setting were included only in the models for major complications and any complications and were therefore not uniformly available across all outcomes. Including all predictors in the main figure would have increased visual complexity and reduced readability, without facilitating direct comparison across prediction targets. We therefore focused the main figure on predictors shared across the broader set of models, while the complete variable-importance results for all predictors are provided in the Supplementary Material to ensure transparency and reproducibility.

## 4. Discussion

This pilot study compared several machine learning algorithms for predicting overall and specific postoperative adverse events and recovery outcomes, with the aim of supporting individualized risk assessment and targeted interventions. Model performance was outcome-specific: logistic regression was selected as the best-performing algorithm for six of the seven prediction targets, whereas SVM was selected for POI. Nevertheless, no algorithm consistently performed best across all outcomes, and the more complex models did not demonstrate a clear overall advantage over logistic regression. Models incorporating preoperative, perioperative, and postoperative features generally achieved the strongest predictive performance, suggesting that the availability of relevant information across the care pathway may be more influential than algorithmic complexity alone. These findings emphasize the importance of high-quality data and clinically meaningful predictors when developing useful perioperative prediction models.

The limited performance indicates that the main challenge may not only lie in the model selection, but also in complex relationships underlying the clinical variables, particularly if these relationships are highly non-linear, time-dependent, or driven by unmeasured biological and clinical factors. Therefore, we suggest that future work on improving ML-based risk prediction strategies should focus on data quality, integrating dynamic perioperative and postoperative measurements, exploring more flexible modeling frameworks, and validating model performance in larger external cohorts.

Given the limited performance across all selected models, SHAP analysis was used to improve the interpretability of the predictions to better understand the contributions of each clinical variable. SHAP analysis can help identify which peri- or postoperative variables contributed to what extent to model predictions and whether their effects were consistent with clinical expectations. Although SHAP does not indicate causality, it provides useful insights into the explainability of the predictions, the prioritization of predictor variables, and the identification of modifiable clinical factors.

A multitude of predictive scores and risk stratification models are commonly used in our institution to stratify individual risk profiles. These repeatedly validated tools are based on different risk factors to anticipate postoperative morbid-mortality and comprise the American Society of Anesthesiologists (ASA) score, the American College of Surgeons Surgical Risk Calculator, and the Physiologic and Operative Severity Score for the Enumeration of Mortality and Morbidity (POSSUM) score [14,15,16]. More recent ML methods display promising clinical utility and applicability in accurately predicting the risk of complications following colorectal surgery according to a recently published meta-analysis, emerging as alternatives to linear prediction models [17]. However, given limited experience and validation, they should still be considered as an adjunct while further validation studies are underway [18]. The aim of this present study was thus to evaluate risk prediction with ML input in the busy clinical environment of our tertiary colorectal referral center (all-comers) to potentially replace conventional tools in the future. To apply these findings to our institutional data, the present pilot study thus focused on demonstrating and developing ML-driven methodological aspects rather than comprehensive and exhaustive clinical risk prediction, including only a limited number of confounders per outcome of interest. Nevertheless, SHAP analysis ultimately enabled a quantitative appraisal of feature importance (Figure 2).

The patterns are interesting and not only confirm the major impact of perioperative management on postoperative outcomes but also help quantify the impact of the individual confounders. Unsurprisingly, perioperative contamination as a surrogate of advanced disease presentation and surgical difficulty was a major predictor of overall and major complications. The analysis further revealed fluid overload and weight gain as potent predictors of complications and delayed recovery, including respiratory complications, POI and postoperative mobilization, in line with findings of former institutional series [8,19]. Respiratory complications further strongly correlated with advanced age, decreased postoperative mobilization, and decreased oral fluid intake on POD2 as a surrogate of delayed functional recovery. Interestingly, renal dysfunction was most strongly linked to fluid overload, decreased mobilization and postoperative weight gain, further supporting the importance of stringent fluid management in colorectal surgery [20,21]. Importantly, despite the fact that most complications after colorectal surgery occur after 48 h, some of the predictors may be the consequence of early complications rather than predicting them (i.e., decreased mobilization or weight gain at POD2) [22]. On the other hand, the strong impact of recovery variables such as postoperative mobilization, weight and oral intake emphasizes the importance of tailored ERAS pathways to refine and improve perioperative recovery trajectories [3].

From the clinical perspective, the present pilot study aligns with former AI-driven work from our group to refine perioperative pathways to individualized, tailored patient care for best possible recovery and outcomes [3]. A next step may incorporate the development of risk scores or calculators to translate the findings into applications for clinical practice.

This study has several limitations. First, the list of confounders was intentionally limited to only few data items through selective feature assignment, leading to a potential risk of outcome-specific overfitting. Two outcomes (any and major complications) were more exhaustively assessed including further data items as suggested by previous ML-based models [23], but overall, the study clearly focused on methodological aspects rather than including a comprehensive list of potential risk factors. Clinical applicability and general validity of the resulting models thus need further evaluation, since potentially relevant predictors may not have been considered in the pilot setting with limited feature selection of the present study. Second, even though training and validation sets were defined, the study lacks external validation. Third, the present study did not formally compare the results to a clinical baseline such as established scores, mainly due to the focus on ML methodology rather than clinical considerations. In a next step, we will evaluate whether and how the present findings can help advance risk prediction in our institution.

## 5. Conclusions

Complex models in the setting of a structured and limited perioperative dataset only slightly outperform simpler approaches, highlighting the importance of data quality and feature richness rather than just increased model complexity. Utility in clinical practice needs to be further demonstrated by larger scale studies with more comprehensive data.

## Figures and Tables

**Table 1:** Prediction targets and corresponding predictors. POD—postoperative day; BMI—body mass index; IV—intravenous; POI—postoperative ileus; ASA—American Society of Anesthesiologists.

Prediction Target	Predictors
Mobilization on POD 2	Age BMI Preoperative weight change (kg) Weight change on POD 2 (kg) Total IV volume of fluids intraoperatively (mL) Oral fluids, total volume taken on POD 1 (mL)
Infectious Complication	Length of incision (cm) Oral fluids, total volume taken on POD 2 (mL) Mobilization on POD 2 Weight change on POD 2 (kg)
Respiratory Complication	Age Smoking Oral fluids, total volume taken on POD 2 (mL) Mobilization on POD 2 Weight change POD 2 (kg)
Major Complication	Age Peritoneal soiling/contamination Oral fluids, total volume taken on POD 2 (mL) Mobilization on POD 2 Weight change on POD 2 (kg) Diabetes Surgical setting Surgical approach ASA physical status
Any Complication	Age Peritoneal soiling/contamination Oral fluids, total volume taken on POD 2 (mL) Mobilization on POD 2 Weight change on POD 2 (kg) Diabetes Surgical setting Surgical approach ASA physical status
Nasogastric Tube Reinsertion (POI)	Age BMI Oral fluids, total volume taken—on postoperative day 2 (mL) Mobilization on POD 2 Weight change on POD 2 (kg) Total IV volume of fluids POD 0 (mL)
Renal Dysfunction	Oral fluids, total volume taken on POD 2 (mL) Mobilization on POD 2 Weight change on POD 2 (kg) Total IV volume of fluids on POD 0 (mL)

**Table 2:** Std—standard deviation; BMI—body mass index; POD—postoperative day.

Variable	Percentage of Missing Values	Mean ± Std or Percentage
Age	0	62.18 ± 16.97
BMI	0.87%	25.47 ± 5.16
Total IV volume of fluids intraoperatively (mL)	0	1839.70 ± 1148.84
Weight change on POD 2 (kg)	30.85%	1.70 ± 3.14
Preoperative weight change (kg)	51.19%	−1.37 ± 4.96
Mobilization on POD 2	22.30%	60.21%
Oral fluids, total volume taken on POD 1 (mL)	18.83%	1499.01 ± 740.07
Oral fluids, total volume taken on POD 2 (mL)	23.39%	1469 ± 780.22
Peritoneal soiling/contamination	0.36%	39.39%
Smoking	0	22.52%
Length of incision (cm)	21.80%	13.46 ± 10.19
Total IV volume of fluids on POD 0 (mL)	0	2817.34 ± 1456.76
Diabetes	0	87.98%
ASA physical status	0.07%	1: 5.65% 2: 64.86% 3: 27.90% 4: 1.59%
Surgery type	0	Elective: 80.59% Emergency: 19.41%
Surgical approach	0	Open: 45.91% MIS: 54.09%

**Table 3:** ROC-AUC—receiver operating characteristics–area under the curve; POD—postoperative day; SSI—surgical site infection; POI—postoperative ileus; SVM—support vector machine. Values are presented with the mean and standard deviation of 5-fold cross-validation.

Target	Best Performance Model	F1-Score	Precision	Recall (Sensitivity)	Specificity	ROC-AUC	ROC-AUC Baseline-Random Guess
Any Complication	Logistic Regression	0.593 ± 0.048	0.506 ± 0.080	0.743 ± 0.100	0.878 ± 0.050	0.844 ± 0.043	0.48
Major Complication	Logistic Regression	0.400 ± 0.118	0.358 ± 0.194	0.618 ± 0.214	0.943 ± 0.066	0.891 ± 0.063	0.48
SSI	Logistic Regression	0.546 ± 0.068	0.465 ± 0.112	0.697 ± 0.085	0.811 ± 0.089	0.787 ± 0.051	0.49
Respiratory Complication	Logistic Regression	0.441 ± 0.125	0.440 ± 0.203	0.513 ± 0.164	0.910 ± 0.082	0.743 ± 0.084	0.50
Mobilization POD2	Logistic Regression	0.820 ± 0.022	0.709 ± 0.052	0.978 ± 0.039	0.192 ± 0.198	0.638 ± 0.078	0.45
Renal Dysfunction	Logistic Regression	0.381 ± 0.152	0.459 ± 0.319	0.488 ± 0.220	0.948 ± 0.077	0.807 ± 0.075	0.48
POI	SVM	0.626 ± 0.116	0.677 ± 0.180	0.627 ± 0.174	0.921 ± 0.059	0.782 ± 0.100	0.41

## Data Availability

The raw data supporting the conclusions of this article will be made available by the authors on request.

## Supplementary Materials

The following supporting information can be downloaded at: https://www.mdpi.com/article/10.3390/diagnostics16193190/s1, Supplementary information including Model Configuration and Hyperparameters, Comparison on different sampling strategy, Comparison on pre, peri and postoperative predictors, Final model comparison, SHAP feature rankings for all variables.
