# Supplementary material for: Machine Learning for Risk Prediction in Colorectal Surgery—Game Changer or Unnecessary? An Institutional Pilot Study
Source: Diagnostics (Basel). 2026 Oct 1;16(19):3190. doi: 10.3390/diagnostics16193190 (PMC13664544; doi:10.3390/diagnostics16193190)
Supplement: Supplementary file 1 [file diagnostics-16-03190-s001.zip › diagnostics-4378858-supplementary.pdf]

Supplementary materials

Model Configurations and Hyperparameters

Model	Base Configuration	Hyperparameters Tuned	Search Values
**Logistic Regression**	max_iter=1000, class_weight='balanced'	C	0.1, 1, 10
		solver	lbfgs, liblinear
**Decision Tree**	class_weight='balanced', random_state=random_state	max_depth	None, 5, 10
		min_samples_split	2, 5
**Random Forest**	class_weight='balanced', random_state=random_state	n_estimators	50, 100
		max_depth	None, 10
**Support Vector Machine (SVM)**	probability=True, class_weight='balanced', random_state=random_state	C	0.1, 1
		kernel	linear, rbf
**Gradient Boosting**	random_state=random_state	n_estimators	50, 100
		learning_rate	0.01, 0.1
		max_depth	3, 5
**XGBoost**	eval_metric='logloss', random_state=random_state, n_jobs=-1	n_estimators	50, 100
		learning_rate	0.01, 0.1
		max_depth	3, 5

Model	Hyperparameter Grid
Logistic Regression	C ∈ {0.1, 1, 10}; solver ∈ {lbfgs, liblinear}
Decision Tree	max_depth ∈ {None, 5, 10}; min_samples_split ∈ {2, 5}

Random Forest	n_estimators ∈ {50, 100};<br>max_depth ∈ {None, 10}
SVM	C ∈ {0.1, 1}; kernel ∈ {linear, rbf}
Gradient Boosting	n_estimators ∈ {50, 100};<br>learning_rate ∈ {0.01, 0.1};<br>max_depth ∈ {3, 5}
XGBoost	n_estimators ∈ {50, 100};<br>learning_rate ∈ {0.01, 0.1};<br>max_depth ∈ {3, 5}

Collinearity Analysis

- Pearson correlation on numerical predictors

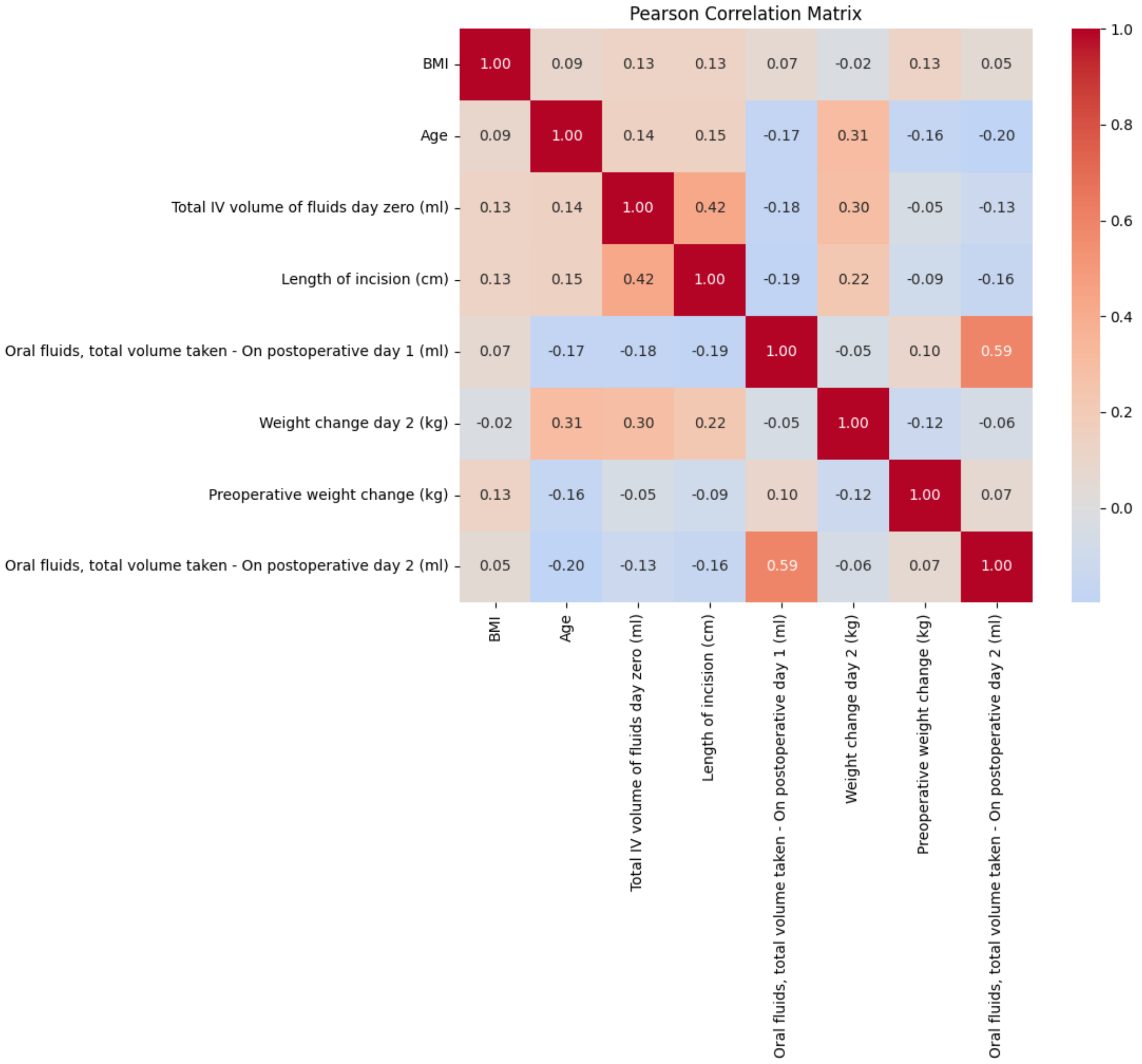

- VIF on numerical predictors

Variable	VIF
Length of incision (cm)	1.47104946
Oral fluids, total volume taken - On postoperative day 2 (ml)	1.41009813
Total IV volume of fluids day zero (ml)	1.40765604
Oral fluids, total volume taken - On postoperative day 1 (ml)	1.39813902
Age	1.23384759
Weight change day 2 (kg)	1.20382857
BMI	1.15224437
Preoperative weight change (kg)	1.04769664

- Cramer's V on categorical predictors

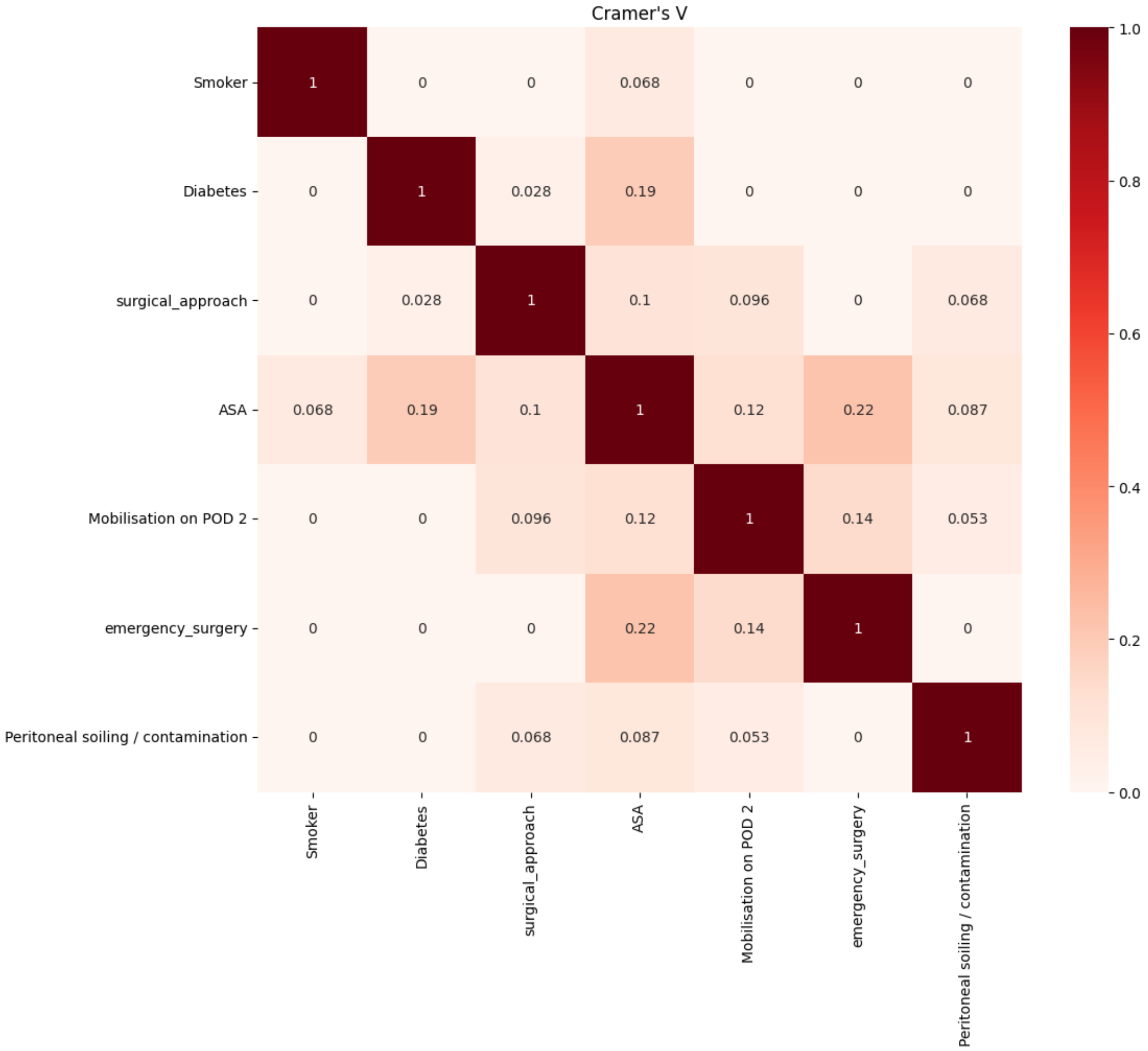

Comparison on different sampling strategy

RAW: no sampling

BALANCED: sampling using the class weight

SMOTE: sampling using SMOTE

Major Complications

**Model (BALANCED)**	**F1 Score**	**Precision**	**Recall**	**Sensitivity**	**Specificity**	**ROC-AUC**
LogisticRegression	0.400 ± 0.118	0.358 ± 0.194	0.618 ± 0.214	0.618 ± 0.214	0.943 ± 0.066	0.891 ± 0.063
GradientBoosting	0.386 ± 0.136	0.323 ± 0.198	0.658 ± 0.185	0.658 ± 0.185	0.927 ± 0.062	0.875 ± 0.095
XGBoost	0.365 ± 0.135	0.319 ± 0.202	0.602 ± 0.222	0.602 ± 0.222	0.925 ± 0.084	0.873 ± 0.072
SVM	0.351 ± 0.104	0.317 ± 0.231	0.636 ± 0.231	0.636 ± 0.231	0.922 ± 0.067	0.873 ± 0.059
RandomForest	0.342 ± 0.117	0.319 ± 0.247	0.604 ± 0.215	0.604 ± 0.215	0.926 ± 0.058	0.840 ± 0.093
DecisionTree	0.275 ± 0.131	0.204 ± 0.123	0.616 ± 0.246	0.616 ± 0.246	0.812 ± 0.309	0.736 ± 0.150

**Model (RAW)**	**F1 Score**	**Precision**	**Recall**	**Sensitivity**	**Specificity**	**ROC-AUC**
LogisticRegression	0.413 ± 0.130	0.379 ± 0.231	0.654 ± 0.210	0.654 ± 0.210	0.937 ± 0.068	0.894 ± 0.058
GradientBoosting	0.386 ± 0.136	0.323 ± 0.198	0.658 ± 0.185	0.658 ± 0.185	0.927 ± 0.062	0.875 ± 0.095
XGBoost	0.365 ± 0.135	0.319 ± 0.202	0.602 ± 0.222	0.602 ± 0.222	0.925 ± 0.084	0.873 ± 0.072
RandomForest	0.362 ± 0.113	0.299 ± 0.148	0.674 ± 0.237	0.674 ± 0.237	0.920 ± 0.066	0.866 ± 0.078
SVM	0.307 ± 0.127	0.340 ± 0.304	0.686 ± 0.303	0.686 ± 0.303	0.844 ± 0.207	0.817 ± 0.156

DecisionTree	0.287 ± 0.156	0.238 ± 0.179	0.586 ± 0.282	0.586 ± 0.282	0.816 ± 0.315	0.739 ± 0.153

**Model (RAW)**	**F1 Score**	**Precision**	**Recall**	**Sensitivity**	**Specificity**	**ROC-AUC**
LogisticRegression	0.413 ± 0.130	0.379 ± 0.231	0.654 ± 0.210	0.654 ± 0.210	0.937 ± 0.068	0.894 ± 0.058
GradientBoosting	0.386 ± 0.136	0.323 ± 0.198	0.658 ± 0.185	0.658 ± 0.185	0.927 ± 0.062	0.875 ± 0.095
XGBoost	0.365 ± 0.135	0.319 ± 0.202	0.602 ± 0.222	0.602 ± 0.222	0.925 ± 0.084	0.873 ± 0.072
RandomForest	0.362 ± 0.113	0.299 ± 0.148	0.674 ± 0.237	0.674 ± 0.237	0.920 ± 0.066	0.866 ± 0.078
SVM	0.307 ± 0.127	0.340 ± 0.304	0.686 ± 0.303	0.686 ± 0.303	0.844 ± 0.207	0.817 ± 0.156
DecisionTree	0.287 ± 0.156	0.238 ± 0.179	0.586 ± 0.282	0.586 ± 0.282	0.816 ± 0.315	0.739 ± 0.153

Any Complications

**Model (BALANCED)**	**F1 Score**	**Precision**	**Recall**	**Sensitivity**	**Specificity**	**ROC-AUC**
LogisticRegression	0.593 ± 0.048	0.506 ± 0.080	0.743 ± 0.100	0.743 ± 0.100	0.878 ± 0.050	0.844 ± 0.043
XGBoost	0.588 ± 0.051	0.554 ± 0.098	0.650 ± 0.098	0.650 ± 0.098	0.910 ± 0.040	0.835 ± 0.038
GradientBoosting	0.572 ± 0.039	0.527 ± 0.104	0.666 ± 0.117	0.666 ± 0.117	0.895 ± 0.050	0.837 ± 0.042
RandomForest	0.564 ± 0.059	0.502 ± 0.089	0.669 ± 0.109	0.669 ± 0.109	0.890 ± 0.038	0.824 ± 0.045
SVM	0.563 ± 0.045	0.509 ± 0.127	0.699 ± 0.141	0.699 ± 0.141	0.875 ± 0.066	0.834 ± 0.037
DecisionTree	0.501 ± 0.076	0.478 ± 0.104	0.552 ± 0.105	0.552 ± 0.105	0.896 ± 0.051	0.726 ± 0.064

**Model (RAW)**	**F1 Score**	**Precision**	**Recall**	**Sensitivity**	**Specificity**	**ROC-AUC**
LogisticRegression	0.593 ± 0.054	0.502 ± 0.086	0.754 ± 0.093	0.754 ± 0.093	0.873 ± 0.052	0.846 ± 0.043
XGBoost	0.588 ± 0.051	0.554 ± 0.098	0.650 ± 0.098	0.650 ± 0.098	0.910 ± 0.040	0.835 ± 0.038

RandomForest	0.578 ± 0.050	0.513 ± 0.090	0.686 ± 0.091	0.686 ± 0.091	0.889 ± 0.048	0.832 ± 0.039
GradientBoosting	0.572 ± 0.039	0.527 ± 0.104	0.666 ± 0.117	0.666 ± 0.117	0.895 ± 0.050	0.837 ± 0.042
SVM	0.524 ± 0.065	0.468 ± 0.114	0.646 ± 0.138	0.646 ± 0.138	0.870 ± 0.064	0.799 ± 0.051
DecisionTree	0.484 ± 0.070	0.426 ± 0.121	0.611 ± 0.136	0.611 ± 0.136	0.855 ± 0.062	0.738 ± 0.078

**Model (RAW)**	**F1 Score**	**Precision**	**Recall**	**Sensitivity**	**Specificity**	**ROC-AUC**
LogisticRegression	0.593 ± 0.054	0.502 ± 0.086	0.754 ± 0.093	0.754 ± 0.093	0.873 ± 0.052	0.846 ± 0.043
XGBoost	0.588 ± 0.051	0.554 ± 0.098	0.650 ± 0.098	0.650 ± 0.098	0.910 ± 0.040	0.835 ± 0.038
RandomForest	0.578 ± 0.050	0.513 ± 0.090	0.686 ± 0.091	0.686 ± 0.091	0.889 ± 0.048	0.832 ± 0.039
GradientBoosting	0.572 ± 0.039	0.527 ± 0.104	0.666 ± 0.117	0.666 ± 0.117	0.895 ± 0.050	0.837 ± 0.042
SVM	0.524 ± 0.065	0.468 ± 0.114	0.646 ± 0.138	0.646 ± 0.138	0.870 ± 0.064	0.799 ± 0.051
DecisionTree	0.484 ± 0.070	0.426 ± 0.121	0.611 ± 0.136	0.611 ± 0.136	0.855 ± 0.062	0.738 ± 0.078

SSI

**Model (BALANCED)**	**F1 Score**	**Precision**	**Recall**	**Sensitivity**	**Specificity**	**ROC-AUC**
LogisticRegression	0.546 ± 0.068	0.465 ± 0.112	0.697 ± 0.085	0.697 ± 0.085	0.811 ± 0.089	0.787 ± 0.051
SVM	0.535 ± 0.065	0.446 ± 0.101	0.708 ± 0.104	0.708 ± 0.104	0.795 ± 0.097	0.781 ± 0.057
XGBoost	0.513 ± 0.063	0.447 ± 0.079	0.641 ± 0.136	0.641 ± 0.136	0.819 ± 0.080	0.763 ± 0.051
GradientBoosting	0.498 ± 0.067	0.419 ± 0.096	0.667 ± 0.135	0.667 ± 0.135	0.774 ± 0.151	0.758 ± 0.053
RandomForest	0.472 ± 0.053	0.407 ± 0.123	0.635 ± 0.140	0.635 ± 0.140	0.767 ± 0.142	0.739 ± 0.052
DecisionTree	0.425 ± 0.068	0.352 ± 0.102	0.600 ± 0.150	0.600 ± 0.150	0.725 ± 0.189	0.668 ± 0.069

Model (RAW)	F1 Score	Precision	Recall	Sensitivity	Specificity	ROC-AUC
LogisticRegression	0.542 ± 0.068	0.472 ± 0.108	0.680 ± 0.105	0.680 ± 0.105	0.818 ± 0.101	0.786 ± 0.052
XGBoost	0.513 ± 0.063	0.447 ± 0.079	0.641 ± 0.136	0.641 ± 0.136	0.819 ± 0.080	0.763 ± 0.051
GradientBoosting	0.498 ± 0.067	0.419 ± 0.096	0.667 ± 0.135	0.667 ± 0.135	0.774 ± 0.151	0.758 ± 0.053
RandomForest	0.474 ± 0.062	0.444 ± 0.137	0.602 ± 0.173	0.602 ± 0.173	0.791 ± 0.156	0.735 ± 0.063
DecisionTree	0.460 ± 0.057	0.401 ± 0.147	0.624 ± 0.147	0.624 ± 0.147	0.767 ± 0.108	0.712 ± 0.057
SVM	0.416 ± 0.090	0.364 ± 0.151	0.663 ± 0.226	0.663 ± 0.226	0.618 ± 0.348	0.585 ± 0.186

Model (SMOTE)	F1 Score	Precision	Recall	Sensitivity	Specificity	ROC-AUC
LogisticRegression	0.542 ± 0.069	0.467 ± 0.112	0.691 ± 0.100	0.691 ± 0.100	0.809 ± 0.110	0.787 ± 0.049
SVM	0.537 ± 0.064	0.445 ± 0.100	0.713 ± 0.092	0.713 ± 0.092	0.794 ± 0.095	0.785 ± 0.050
XGBoost	0.506 ± 0.060	0.440 ± 0.106	0.659 ± 0.152	0.659 ± 0.152	0.798 ± 0.100	0.764 ± 0.050
GradientBoosting	0.497 ± 0.054	0.421 ± 0.113	0.695 ± 0.167	0.695 ± 0.167	0.757 ± 0.152	0.762 ± 0.045
RandomForest	0.473 ± 0.053	0.399 ± 0.122	0.678 ± 0.166	0.678 ± 0.166	0.742 ± 0.143	0.733 ± 0.050
DecisionTree	0.443 ± 0.059	0.337 ± 0.080	0.720 ± 0.149	0.720 ± 0.149	0.665 ± 0.159	0.703 ± 0.064

Respiratory Complications

Model (BALANCED)	F1 Score	Precision	Recall	Sensitivity	Specificity	ROC-AUC
LogisticRegression	0.441 ± 0.125	0.440 ± 0.203	0.513 ± 0.164	0.513 ± 0.164	0.910 ± 0.082	0.743 ± 0.084
SVM	0.419 ± 0.106	0.370 ± 0.149	0.590 ± 0.156	0.590 ± 0.156	0.859 ± 0.116	0.767 ± 0.078
RandomForest	0.377 ± 0.066	0.336 ± 0.148	0.573 ± 0.203	0.573 ± 0.203	0.845 ± 0.112	0.754 ± 0.052

XGBoost	0.370 ± 0.083	0.300 ± 0.110	0.613 ± 0.203	0.613 ± 0.203	0.816 ± 0.137	0.751 ± 0.069
GradientBoosting	0.369 ± 0.074	0.273 ± 0.083	0.643 ± 0.151	0.643 ± 0.151	0.810 ± 0.087	0.756 ± 0.066
DecisionTree	0.278 ± 0.063	0.234 ± 0.093	0.473 ± 0.229	0.473 ± 0.229	0.792 ± 0.205	0.620 ± 0.089

**Model (RAW)**	**F1 Score**	**Precision**	**Recall**	**Sensitivity**	**Specificity**	**ROC-AUC**
LogisticRegression	0.438 ± 0.118	0.427 ± 0.164	0.510 ± 0.181	0.510 ± 0.181	0.914 ± 0.077	0.751 ± 0.081
RandomForest	0.374 ± 0.073	0.316 ± 0.128	0.543 ± 0.136	0.543 ± 0.136	0.856 ± 0.085	0.754 ± 0.057
XGBoost	0.370 ± 0.083	0.300 ± 0.110	0.613 ± 0.203	0.613 ± 0.203	0.816 ± 0.137	0.751 ± 0.069
GradientBoosting	0.369 ± 0.074	0.273 ± 0.083	0.643 ± 0.151	0.643 ± 0.151	0.810 ± 0.087	0.756 ± 0.066
SVM	0.305 ± 0.086	0.311 ± 0.176	0.473 ± 0.234	0.473 ± 0.234	0.801 ± 0.233	0.617 ± 0.134
DecisionTree	0.288 ± 0.077	0.257 ± 0.095	0.463 ± 0.233	0.463 ± 0.233	0.803 ± 0.220	0.650 ± 0.091

**Model (SMOTE)**	**F1 Score**	**Precision**	**Recall**	**Sensitivity**	**Specificity**	**ROC-AUC**
LogisticRegression	0.440 ± 0.123	0.450 ± 0.180	0.487 ± 0.163	0.487 ± 0.163	0.919 ± 0.087	0.741 ± 0.086
SVM	0.383 ± 0.099	0.314 ± 0.128	0.610 ± 0.184	0.610 ± 0.184	0.821 ± 0.141	0.745 ± 0.083
XGBoost	0.368 ± 0.082	0.297 ± 0.116	0.613 ± 0.185	0.613 ± 0.185	0.818 ± 0.109	0.737 ± 0.080
GradientBoosting	0.361 ± 0.083	0.296 ± 0.125	0.583 ± 0.177	0.583 ± 0.177	0.826 ± 0.105	0.733 ± 0.080
RandomForest	0.352 ± 0.064	0.287 ± 0.101	0.570 ± 0.192	0.570 ± 0.192	0.824 ± 0.128	0.735 ± 0.058
DecisionTree	0.311 ± 0.084	0.245 ± 0.089	0.490 ± 0.153	0.490 ± 0.153	0.825 ± 0.103	0.665 ± 0.096

Renal Dysfunction

Model (BALANCED)	F1 Score	Precision	Recall	Sensitivity	Specificity	ROC-AUC
LogisticRegression	0.381 ± 0.152	0.459 ± 0.319	0.488 ± 0.220	0.488 ± 0.220	0.948 ± 0.077	0.807 ± 0.075
XGBoost	0.340 ± 0.100	0.299 ± 0.186	0.580 ± 0.247	0.580 ± 0.247	0.936 ± 0.047	0.861 ± 0.062
RandomForest	0.320 ± 0.113	0.389 ± 0.324	0.472 ± 0.258	0.472 ± 0.258	0.948 ± 0.045	0.793 ± 0.108
GradientBoosting	0.308 ± 0.089	0.383 ± 0.331	0.498 ± 0.237	0.498 ± 0.237	0.931 ± 0.070	0.839 ± 0.070
SVM	0.285 ± 0.075	0.274 ± 0.243	0.626 ± 0.285	0.626 ± 0.285	0.896 ± 0.080	0.828 ± 0.073
DecisionTree	0.186 ± 0.122	0.229 ± 0.277	0.508 ± 0.350	0.508 ± 0.350	0.650 ± 0.456	0.573 ± 0.100

Model (RAW)	F1 Score	Precision	Recall	Sensitivity	Specificity	ROC-AUC
LogisticRegression	0.372 ± 0.142	0.469 ± 0.312	0.458 ± 0.222	0.458 ± 0.222	0.956 ± 0.058	0.813 ± 0.075
XGBoost	0.340 ± 0.100	0.299 ± 0.186	0.580 ± 0.247	0.580 ± 0.247	0.936 ± 0.047	0.861 ± 0.062
GradientBoosting	0.308 ± 0.089	0.383 ± 0.331	0.498 ± 0.237	0.498 ± 0.237	0.931 ± 0.070	0.839 ± 0.070
RandomForest	0.302 ± 0.081	0.340 ± 0.244	0.452 ± 0.242	0.452 ± 0.242	0.944 ± 0.049	0.782 ± 0.112
SVM	0.277 ± 0.123	0.289 ± 0.208	0.476 ± 0.262	0.476 ± 0.262	0.841 ± 0.298	0.615 ± 0.195
DecisionTree	0.232 ± 0.088	0.243 ± 0.212	0.452 ± 0.269	0.452 ± 0.269	0.876 ± 0.202	0.702 ± 0.123

Model (SMOTE)	F1 Score	Precision	Recall	Sensitivity	Specificity	ROC-AUC
LogisticRegression	0.370 ± 0.153	0.506 ± 0.353	0.468 ± 0.241	0.468 ± 0.241	0.946 ± 0.083	0.798 ± 0.076
GradientBoosting	0.339 ± 0.134	0.418 ± 0.329	0.528 ± 0.241	0.528 ± 0.241	0.923 ± 0.089	0.833 ± 0.075
RandomForest	0.328 ± 0.102	0.335 ± 0.281	0.640 ± 0.277	0.640 ± 0.277	0.905 ± 0.097	0.834 ± 0.088
XGBoost	0.328 ± 0.119	0.286 ± 0.218	0.626 ± 0.200	0.626 ± 0.200	0.909 ± 0.064	0.836 ± 0.078

SVM	0.267 ± 0.115	0.239 ± 0.212	0.612 ± 0.298	0.612 ± 0.298	0.864 ± 0.143	0.777 ± 0.091
DecisionTree	0.235 ± 0.130	0.207 ± 0.207	0.518 ± 0.241	0.518 ± 0.241	0.828 ± 0.259	0.702 ± 0.141

POI

Model (BALANCED)	F1 Score	Precision	Recall	Sensitivity	Specificity	ROC-AUC
SVM	0.626 ± 0.116	0.677 ± 0.180	0.627 ± 0.174	0.627 ± 0.174	0.921 ± 0.059	0.782 ± 0.100
LogisticRegression	0.620 ± 0.125	0.617 ± 0.184	0.691 ± 0.172	0.691 ± 0.172	0.867 ± 0.172	0.776 ± 0.095
GradientBoosting	0.612 ± 0.144	0.693 ± 0.228	0.611 ± 0.166	0.611 ± 0.166	0.896 ± 0.167	0.743 ± 0.085
XGBoost	0.586 ± 0.143	0.704 ± 0.249	0.594 ± 0.200	0.594 ± 0.200	0.885 ± 0.181	0.749 ± 0.087
RandomForest	0.585 ± 0.121	0.656 ± 0.231	0.594 ± 0.152	0.594 ± 0.152	0.899 ± 0.097	0.775 ± 0.094
DecisionTree	0.459 ± 0.125	0.506 ± 0.234	0.581 ± 0.248	0.581 ± 0.248	0.726 ± 0.376	0.636 ± 0.102

Model (RAW)	F1 Score	Precision	Recall	Sensitivity	Specificity	ROC-AUC
LogisticRegression	0.625 ± 0.132	0.668 ± 0.181	0.637 ± 0.186	0.637 ± 0.186	0.913 ± 0.085	0.772 ± 0.098
RandomForest	0.625 ± 0.131	0.639 ± 0.197	0.643 ± 0.120	0.643 ± 0.120	0.902 ± 0.081	0.784 ± 0.080
GradientBoosting	0.612 ± 0.144	0.693 ± 0.228	0.611 ± 0.166	0.611 ± 0.166	0.896 ± 0.167	0.743 ± 0.085
SVM	0.597 ± 0.121	0.629 ± 0.193	0.652 ± 0.207	0.652 ± 0.207	0.879 ± 0.129	0.751 ± 0.095
XGBoost	0.586 ± 0.143	0.704 ± 0.249	0.594 ± 0.200	0.594 ± 0.200	0.885 ± 0.181	0.749 ± 0.087
DecisionTree	0.514 ± 0.137	0.499 ± 0.198	0.636 ± 0.202	0.636 ± 0.202	0.767 ± 0.301	0.689 ± 0.113

Model (SMOTE)	F1 Score	Precision	Recall	Sensitivity	Specificity	ROC-AUC

SVM	0.622 ± 0.116	0.658 ± 0.198	0.634 ± 0.148	0.634 ± 0.148	0.910 ± 0.071	0.777 ± 0.094
LogisticRegression	0.620 ± 0.128	0.642 ± 0.211	0.673 ± 0.166	0.673 ± 0.166	0.876 ± 0.156	0.774 ± 0.090
XGBoost	0.589 ± 0.144	0.651 ± 0.241	0.606 ± 0.167	0.606 ± 0.167	0.889 ± 0.123	0.747 ± 0.085
RandomForest	0.580 ± 0.120	0.565 ± 0.199	0.669 ± 0.160	0.669 ± 0.160	0.853 ± 0.109	0.761 ± 0.091
GradientBoosting	0.546 ± 0.109	0.590 ± 0.244	0.609 ± 0.182	0.609 ± 0.182	0.852 ± 0.151	0.717 ± 0.094
DecisionTree	0.456 ± 0.103	0.425 ± 0.156	0.572 ± 0.171	0.572 ± 0.171	0.766 ± 0.250	0.646 ± 0.071

Mobilisation POD2

Model (BALANCED)	F1 Score	Precision	Recall	Sensitivity	Specificity	ROC-AUC
LogisticRegression	0.820 ± 0.022	0.709 ± 0.052	0.978 ± 0.039	0.978 ± 0.039	0.192 ± 0.198	0.638 ± 0.078
SVM	0.812 ± 0.012	0.702 ± 0.046	0.970 ± 0.051	0.970 ± 0.051	0.173 ± 0.174	0.623 ± 0.113
XGBoost	0.812 ± 0.013	0.698 ± 0.040	0.976 ± 0.047	0.976 ± 0.047	0.153 ± 0.170	0.624 ± 0.071
RandomForest	0.811 ± 0.018	0.695 ± 0.042	0.978 ± 0.036	0.978 ± 0.036	0.143 ± 0.167	0.615 ± 0.074
GradientBoosting	0.808 ± 0.014	0.688 ± 0.032	0.981 ± 0.036	0.981 ± 0.036	0.114 ± 0.141	0.624 ± 0.068
DecisionTree	0.800 ± 0.009	0.669 ± 0.019	0.996 ± 0.013	0.996 ± 0.013	0.027 ± 0.076	0.547 ± 0.067

Model (RAW)	F1 Score	Precision	Recall	Sensitivity	Specificity	ROC-AUC
LogisticRegression	0.821 ± 0.022	0.711 ± 0.051	0.977 ± 0.041	0.977 ± 0.041	0.201 ± 0.196	0.639 ± 0.076
SVM	0.813 ± 0.014	0.696 ± 0.037	0.982 ± 0.040	0.982 ± 0.040	0.143 ± 0.149	0.609 ± 0.087
RandomForest	0.812 ± 0.015	0.695 ± 0.043	0.982 ± 0.033	0.982 ± 0.033	0.137 ± 0.161	0.635 ± 0.080
XGBoost	0.812 ± 0.013	0.698 ± 0.040	0.976 ± 0.047	0.976 ± 0.047	0.153 ± 0.170	0.624 ± 0.071
GradientBoosting	0.808 ± 0.014	0.688 ± 0.032	0.981 ± 0.036	0.981 ± 0.036	0.114 ± 0.141	0.624 ± 0.068

DecisionTree	0.799 ± 0.008	0.673 ± 0.032	0.989 ± 0.037	0.989 ± 0.037	0.042 ± 0.133	0.523 ± 0.081

Model (SMOTE)	F1 Score	Precision	Recall	Sensitivity	Specificity	ROC-AUC
LogisticRegression	0.820 ± 0.023	0.710 ± 0.050	0.978 ± 0.038	0.978 ± 0.038	0.195 ± 0.195	0.634 ± 0.076
SVM	0.815 ± 0.017	0.704 ± 0.040	0.974 ± 0.048	0.974 ± 0.048	0.177 ± 0.173	0.619 ± 0.120
RandomForest	0.813 ± 0.014	0.705 ± 0.038	0.966 ± 0.046	0.966 ± 0.046	0.190 ± 0.167	0.606 ± 0.062
XGBoost	0.810 ± 0.017	0.692 ± 0.034	0.981 ± 0.034	0.981 ± 0.034	0.130 ± 0.143	0.612 ± 0.079
GradientBoosting	0.808 ± 0.018	0.692 ± 0.039	0.977 ± 0.037	0.977 ± 0.037	0.130 ± 0.166	0.605 ± 0.074
DecisionTree	0.800 ± 0.012	0.675 ± 0.034	0.987 ± 0.031	0.987 ± 0.031	0.052 ± 0.140	0.539 ± 0.080

Comparison on pre, peri and postoperative predictors

List of pre, peri and postoperative predictors for each prediction target

Pre+peri+postoperative predictors

Target	Predictors
Mobilisation on POD 2	Age; BMI; Preoperative weight change (kg); Weight change day 2 (kg); Total IV volume of fluids day zero (ml); Oral fluids, total volume taken - On postoperative day 1 (ml)
Major complications_flag	Age; Peritoneal soiling / contamination; Oral fluids, total volume taken - On postoperative day 2 (ml); Mobilisation on POD 2; Weight change day 2 (kg); Diabetes; ASA; emergency_surgery; surgical_approach
Respiratory Complications_flag	Age; Smoker; Oral fluids, total volume taken - On postoperative day 2 (ml); Mobilisation on POD 2; Weight change day 2 (kg)

Infectious Complications_flag	Length of incision (cm); Oral fluids, total volume taken - On postoperative day 2 (ml); Mobilisation on POD 2; Weight change day 2 (kg)
Renal dysfunction	Oral fluids, total volume taken - On postoperative day 2 (ml); Mobilisation on POD 2; Weight change day 2 (kg); Total IV volume of fluids day zero (ml)
Nasogastric tube reinserted	Age; BMI; Oral fluids, total volume taken - On postoperative day 2 (ml); Mobilisation on POD 2; Weight change day 2 (kg); Total IV volume of fluids day zero (ml)
Overall complications_flag	Age; Peritoneal soiling / contamination; Oral fluids, total volume taken - On postoperative day 2 (ml); Mobilisation on POD 2; Weight change day 2 (kg); Diabetes; ASA; emergency_surgery; surgical_approach

Pre + Perioperative Predictors

Target	Predictors
Mobilisation on POD 2	Age; BMI; Preoperative weight change (kg); Total IV volume of fluids day zero (ml)
Major complications_flag	Age; Peritoneal soiling / contamination; Diabetes; ASA; emergency_surgery; surgical_approach
Respiratory Complications_flag	Age; Smoker
Infectious Complications_flag	Length of incision (cm); Weight change day 2 (kg)
Renal dysfunction	Total IV volume of fluids day zero (ml)
Nasogastric tube reinserted	Age; BMI; Total IV volume of fluids day zero (ml)

Overall complications_flag	Age; Peritoneal soiling / contamination; Diabetes; ASA; emergency_surgery; surgical_approach

Preoperative predictors

Target	Predictors
Mobilisation on POD 2	Age; BMI; Preoperative weight change (kg)
Major complications_flag	Age; Diabetes; ASA
Respiratory Complications_flag	Age; Smoker
Infectious Complications_flag	None
Renal dysfunction	None
Nasogastric tube reinserted	Age; BMI
Overall complications_flag	Age; Diabetes; ASA

Major Complications

Model (PRE)	F1 Score	Precision	Recall	Sensitivity	Specificity	ROC-AUC
LogisticRegression	0.187 ± 0.031	0.122 ± 0.032	0.497 ± 0.159	0.497 ± 0.159	0.791 ± 0.070	0.600 ± 0.054
XGBoost	0.177 ± 0.042	0.124 ± 0.060	0.495 ± 0.161	0.495 ± 0.161	0.761 ± 0.109	0.593 ± 0.059
GradientBoosting	0.161 ± 0.034	0.098 ± 0.023	0.489 ± 0.127	0.489 ± 0.127	0.743 ± 0.105	0.569 ± 0.063
RandomForest	0.137 ± 0.044	0.156 ± 0.203	0.493 ± 0.341	0.493 ± 0.341	0.609 ± 0.371	0.507 ± 0.080
DecisionTree	0.130 ± 0.034	0.124 ± 0.151	0.555 ± 0.305	0.555 ± 0.305	0.562 ± 0.359	0.501 ± 0.095
SVM	0.124 ± 0.028	0.074 ± 0.029	0.699 ± 0.287	0.699 ± 0.287	0.422 ± 0.332	0.484 ± 0.087

Model (PRE)	F1 Score	Precision	Recall	Sensitivity	Specificity	ROC-AUC
LogisticRegression	0.187 ± 0.031	0.122 ± 0.032	0.497 ± 0.159	0.497 ± 0.159	0.791 ± 0.070	0.600 ± 0.054
XGBoost	0.177 ± 0.042	0.124 ± 0.060	0.495 ± 0.161	0.495 ± 0.161	0.761 ± 0.109	0.593 ± 0.059

GradientBoosting	0.161 ± 0.034	0.098 ± 0.023	0.489 ± 0.127	0.489 ± 0.127	0.743 ± 0.105	0.569 ± 0.063
RandomForest	0.137 ± 0.044	0.156 ± 0.203	0.493 ± 0.341	0.493 ± 0.341	0.609 ± 0.371	0.507 ± 0.080
DecisionTree	0.130 ± 0.034	0.124 ± 0.151	0.555 ± 0.305	0.555 ± 0.305	0.562 ± 0.359	0.501 ± 0.095
SVM	0.124 ± 0.028	0.074 ± 0.029	0.699 ± 0.287	0.699 ± 0.287	0.422 ± 0.332	0.484 ± 0.087

**Model (PRE+PERI+POST)**	**F1 Score**	**Precision**	**Recall**	**Sensitivity**	**Specificity**	**ROC-AUC**
LogisticRegression	0.413 ± 0.130	0.379 ± 0.231	0.654 ± 0.210	0.654 ± 0.210	0.937 ± 0.068	0.894 ± 0.058
GradientBoosting	0.386 ± 0.136	0.323 ± 0.198	0.658 ± 0.185	0.658 ± 0.185	0.927 ± 0.062	0.875 ± 0.095
XGBoost	0.365 ± 0.135	0.319 ± 0.202	0.602 ± 0.222	0.602 ± 0.222	0.925 ± 0.084	0.873 ± 0.072
RandomForest	0.362 ± 0.113	0.299 ± 0.148	0.674 ± 0.237	0.674 ± 0.237	0.920 ± 0.066	0.866 ± 0.078
SVM	0.307 ± 0.127	0.340 ± 0.304	0.686 ± 0.303	0.686 ± 0.303	0.844 ± 0.207	0.817 ± 0.156
DecisionTree	0.287 ± 0.156	0.238 ± 0.179	0.586 ± 0.282	0.586 ± 0.282	0.816 ± 0.315	0.739 ± 0.153

Any Complications

**Model (PRE)**	**F1 Score**	**Precision**	**Recall**	**Sensitivity**	**Specificity**	**ROC-AUC**
LogisticRegression	0.381 ± 0.021	0.272 ± 0.039	0.698 ± 0.169	0.698 ± 0.169	0.494 ± 0.207	0.600 ± 0.034
XGBoost	0.369 ± 0.022	0.256 ± 0.038	0.719 ± 0.152	0.719 ± 0.152	0.438 ± 0.208	0.577 ± 0.040
GradientBoosting	0.365 ± 0.022	0.247 ± 0.031	0.758 ± 0.161	0.758 ± 0.161	0.384 ± 0.216	0.572 ± 0.043
RandomForest	0.362 ± 0.019	0.241 ± 0.027	0.779 ± 0.151	0.779 ± 0.151	0.352 ± 0.198	0.564 ± 0.033
DecisionTree	0.357 ± 0.020	0.243 ± 0.038	0.761 ± 0.200	0.761 ± 0.200	0.349 ± 0.284	0.558 ± 0.041
SVM	0.350 ± 0.017	0.219 ± 0.026	0.924 ± 0.122	0.924 ± 0.122	0.138 ± 0.185	0.486 ± 0.067

Model (PRE+PERI)	F1 Score	Precision	Recall	Sensitivity	Specificity	ROC-AUC
LogisticRegression	0.581 ± 0.035	0.480 ± 0.058	0.758 ± 0.097	0.758 ± 0.097	0.785 ± 0.058	0.807 ± 0.027
GradientBoosting	0.567 ± 0.029	0.460 ± 0.046	0.762 ± 0.105	0.762 ± 0.105	0.767 ± 0.062	0.800 ± 0.025
XGBoost	0.562 ± 0.030	0.445 ± 0.034	0.773 ± 0.078	0.773 ± 0.078	0.753 ± 0.046	0.799 ± 0.023
DecisionTree	0.541 ± 0.028	0.425 ± 0.030	0.760 ± 0.096	0.760 ± 0.096	0.737 ± 0.054	0.767 ± 0.031
SVM	0.519 ± 0.055	0.422 ± 0.071	0.707 ± 0.111	0.707 ± 0.111	0.730 ± 0.167	0.745 ± 0.111
RandomForest	0.506 ± 0.029	0.389 ± 0.046	0.743 ± 0.082	0.743 ± 0.082	0.696 ± 0.077	0.749 ± 0.031

Model (PRE+PERI+POST)	F1 Score	Precision	Recall	Sensitivity	Specificity	ROC-AUC
LogisticRegression	0.593 ± 0.054	0.502 ± 0.086	0.754 ± 0.093	0.754 ± 0.093	0.873 ± 0.052	0.846 ± 0.043
XGBoost	0.588 ± 0.051	0.554 ± 0.098	0.650 ± 0.098	0.650 ± 0.098	0.910 ± 0.040	0.835 ± 0.038
RandomForest	0.578 ± 0.050	0.513 ± 0.090	0.686 ± 0.091	0.686 ± 0.091	0.889 ± 0.048	0.832 ± 0.039
GradientBoosting	0.572 ± 0.039	0.527 ± 0.104	0.666 ± 0.117	0.666 ± 0.117	0.895 ± 0.050	0.837 ± 0.042
SVM	0.524 ± 0.065	0.468 ± 0.114	0.646 ± 0.138	0.646 ± 0.138	0.870 ± 0.064	0.799 ± 0.051
DecisionTree	0.484 ± 0.070	0.426 ± 0.121	0.611 ± 0.136	0.611 ± 0.136	0.855 ± 0.062	0.738 ± 0.078

SSI

Model (PRE+PERI)	F1 Score	Precision	Recall	Sensitivity	Specificity	ROC-AUC
LogisticRegression	0.511 ± 0.047	0.405 ± 0.053	0.709 ± 0.085	0.709 ± 0.085	0.766 ± 0.068	0.772 ± 0.039
XGBoost	0.492 ± 0.047	0.403 ± 0.068	0.653 ± 0.091	0.653 ± 0.091	0.779 ± 0.072	0.761 ± 0.042
GradientBoosting	0.488 ± 0.038	0.393 ± 0.073	0.674 ± 0.084	0.674 ± 0.084	0.758 ± 0.087	0.761 ± 0.040
DecisionTree	0.451 ± 0.043	0.351 ± 0.050	0.654 ± 0.095	0.654 ± 0.095	0.724 ± 0.105	0.718 ± 0.043

RandomForest	0.448 ± 0.038	0.337 ± 0.062	0.717 ± 0.122	0.717 ± 0.122	0.667 ± 0.133	0.717 ± 0.044
SVM	0.428 ± 0.075	0.359 ± 0.115	0.653 ± 0.201	0.653 ± 0.201	0.640 ± 0.332	0.591 ± 0.137

**Model (PRE+PERI+POST)**	**F1 Score**	**Precision**	**Recall**	**Sensitivity**	**Specificity**	**ROC-AUC**
LogisticRegression	0.542 ± 0.068	0.472 ± 0.108	0.680 ± 0.105	0.680 ± 0.105	0.818 ± 0.101	0.786 ± 0.052
XGBoost	0.513 ± 0.063	0.447 ± 0.079	0.641 ± 0.136	0.641 ± 0.136	0.819 ± 0.080	0.763 ± 0.051
GradientBoosting	0.498 ± 0.067	0.419 ± 0.096	0.667 ± 0.135	0.667 ± 0.135	0.774 ± 0.151	0.758 ± 0.053
RandomForest	0.474 ± 0.062	0.444 ± 0.137	0.602 ± 0.173	0.602 ± 0.173	0.791 ± 0.156	0.735 ± 0.063
DecisionTree	0.460 ± 0.057	0.401 ± 0.147	0.624 ± 0.147	0.624 ± 0.147	0.767 ± 0.108	0.712 ± 0.057
SVM	0.416 ± 0.090	0.364 ± 0.151	0.663 ± 0.226	0.663 ± 0.226	0.618 ± 0.348	0.585 ± 0.186

Respiratory Complications

**Model (PRE)**	**F1 Score**	**Precision**	**Recall**	**Sensitivity**	**Specificity**	**ROC-AUC**
LogisticRegression	0.286 ± 0.044	0.211 ± 0.042	0.478 ± 0.115	0.478 ± 0.115	0.802 ± 0.081	0.670 ± 0.040
GradientBoosting	0.279 ± 0.054	0.243 ± 0.060	0.386 ± 0.174	0.386 ± 0.174	0.847 ± 0.147	0.660 ± 0.052
XGBoost	0.277 ± 0.052	0.227 ± 0.068	0.451 ± 0.208	0.451 ± 0.208	0.798 ± 0.180	0.659 ± 0.047
DecisionTree	0.251 ± 0.049	0.217 ± 0.098	0.457 ± 0.220	0.457 ± 0.220	0.754 ± 0.204	0.626 ± 0.060
RandomForest	0.248 ± 0.048	0.231 ± 0.113	0.482 ± 0.278	0.482 ± 0.278	0.715 ± 0.273	0.611 ± 0.051
SVM	0.208 ± 0.034	0.135 ± 0.047	0.729 ± 0.281	0.729 ± 0.281	0.406 ± 0.341	0.521 ± 0.105

**Model (PRE)**	**F1 Score**	**Precision**	**Recall**	**Sensitivity**	**Specificity**	**ROC-AUC**
LogisticRegression	0.286 ± 0.044	0.211 ± 0.042	0.478 ± 0.115	0.478 ± 0.115	0.802 ± 0.081	0.670 ± 0.040
GradientBoosting	0.279 ± 0.054	0.243 ± 0.060	0.386 ± 0.174	0.386 ± 0.174	0.847 ± 0.147	0.660 ± 0.052

XGBoost	0.277 ± 0.052	0.227 ± 0.068	0.451 ± 0.208	0.451 ± 0.208	0.798 ± 0.180	0.659 ± 0.047
DecisionTree	0.251 ± 0.049	0.217 ± 0.098	0.457 ± 0.220	0.457 ± 0.220	0.754 ± 0.204	0.626 ± 0.060
RandomForest	0.248 ± 0.048	0.231 ± 0.113	0.482 ± 0.278	0.482 ± 0.278	0.715 ± 0.273	0.611 ± 0.051
SVM	0.208 ± 0.034	0.135 ± 0.047	0.729 ± 0.281	0.729 ± 0.281	0.406 ± 0.341	0.521 ± 0.105

**Model (PRE+PERI+POST)**	**F1 Score**	**Precision**	**Recall**	**Sensitivity**	**Specificity**	**ROC-AUC**
LogisticRegression	0.438 ± 0.118	0.427 ± 0.164	0.510 ± 0.181	0.510 ± 0.181	0.914 ± 0.077	0.751 ± 0.081
RandomForest	0.374 ± 0.073	0.316 ± 0.128	0.543 ± 0.136	0.543 ± 0.136	0.856 ± 0.085	0.754 ± 0.057
XGBoost	0.370 ± 0.083	0.300 ± 0.110	0.613 ± 0.203	0.613 ± 0.203	0.816 ± 0.137	0.751 ± 0.069
GradientBoosting	0.369 ± 0.074	0.273 ± 0.083	0.643 ± 0.151	0.643 ± 0.151	0.810 ± 0.087	0.756 ± 0.066
SVM	0.305 ± 0.086	0.311 ± 0.176	0.473 ± 0.234	0.473 ± 0.234	0.801 ± 0.233	0.617 ± 0.134
DecisionTree	0.288 ± 0.077	0.257 ± 0.095	0.463 ± 0.233	0.463 ± 0.233	0.803 ± 0.220	0.650 ± 0.091

POI

**Model (PRE)**	**F1 Score**	**Precision**	**Recall**	**Sensitivity**	**Specificity**	**ROC-AUC**
LogisticRegression	0.392 ± 0.026	0.270 ± 0.049	0.785 ± 0.143	0.785 ± 0.143	0.388 ± 0.194	0.560 ± 0.046
XGBoost	0.384 ± 0.023	0.251 ± 0.030	0.858 ± 0.129	0.858 ± 0.129	0.280 ± 0.189	0.516 ± 0.052
GradientBoosting	0.379 ± 0.021	0.246 ± 0.027	0.866 ± 0.113	0.866 ± 0.113	0.255 ± 0.189	0.518 ± 0.047
SVM	0.376 ± 0.022	0.246 ± 0.038	0.874 ± 0.146	0.874 ± 0.146	0.233 ± 0.222	0.500 ± 0.065
RandomForest	0.376 ± 0.021	0.255 ± 0.068	0.853 ± 0.181	0.853 ± 0.181	0.258 ± 0.250	0.516 ± 0.049
DecisionTree	0.366 ± 0.023	0.232 ± 0.029	0.918 ± 0.125	0.918 ± 0.125	0.145 ± 0.205	0.514 ± 0.050

**Model (PRE+PERI)**	**F1 Score**	**Precision**	**Recall**	**Sensitivity**	**Specificity**	**ROC-AUC**

LogisticRegression	0.440 ± 0.042	0.408 ± 0.138	0.582 ± 0.172	0.582 ± 0.172	0.697 ± 0.213	0.629 ± 0.047
RandomForest	0.399 ± 0.036	0.324 ± 0.097	0.662 ± 0.239	0.662 ± 0.239	0.536 ± 0.294	0.569 ± 0.059
GradientBoosting	0.396 ± 0.045	0.310 ± 0.104	0.732 ± 0.245	0.732 ± 0.245	0.432 ± 0.351	0.568 ± 0.067
XGBoost	0.388 ± 0.030	0.295 ± 0.078	0.708 ± 0.235	0.708 ± 0.235	0.447 ± 0.330	0.558 ± 0.058
SVM	0.385 ± 0.034	0.295 ± 0.097	0.753 ± 0.260	0.753 ± 0.260	0.392 ± 0.346	0.526 ± 0.074
DecisionTree	0.372 ± 0.030	0.278 ± 0.083	0.760 ± 0.275	0.760 ± 0.275	0.341 ± 0.384	0.552 ± 0.048

**Model (PRE+PERI+POST)**	**F1 Score**	**Precision**	**Recall**	**Sensitivity**	**Specificity**	**ROC-AUC**
LogisticRegression	0.625 ± 0.132	0.668 ± 0.181	0.637 ± 0.186	0.637 ± 0.186	0.913 ± 0.085	0.772 ± 0.098
RandomForest	0.625 ± 0.131	0.639 ± 0.197	0.643 ± 0.120	0.643 ± 0.120	0.902 ± 0.081	0.784 ± 0.080
GradientBoosting	0.612 ± 0.144	0.693 ± 0.228	0.611 ± 0.166	0.611 ± 0.166	0.896 ± 0.167	0.743 ± 0.085
SVM	0.597 ± 0.121	0.629 ± 0.193	0.652 ± 0.207	0.652 ± 0.207	0.879 ± 0.129	0.751 ± 0.095
XGBoost	0.586 ± 0.143	0.704 ± 0.249	0.594 ± 0.200	0.594 ± 0.200	0.885 ± 0.181	0.749 ± 0.087
DecisionTree	0.514 ± 0.137	0.499 ± 0.198	0.636 ± 0.202	0.636 ± 0.202	0.767 ± 0.301	0.689 ± 0.113

Renal Dysfunction

**Model (PRE+PERI)**	**F1 Score**	**Precision**	**Recall**	**Sensitivity**	**Specificity**	**ROC-AUC**
LogisticRegression	0.239 ± 0.058	0.300 ± 0.190	0.343 ± 0.192	0.343 ± 0.192	0.927 ± 0.087	0.702 ± 0.081
RandomForest	0.236 ± 0.076	0.330 ± 0.248	0.269 ± 0.128	0.269 ± 0.128	0.953 ± 0.053	0.598 ± 0.084
XGBoost	0.191 ± 0.064	0.218 ± 0.154	0.362 ± 0.221	0.362 ± 0.221	0.868 ± 0.143	0.665 ± 0.075
DecisionTree	0.182 ± 0.076	0.277 ± 0.311	0.367 ± 0.239	0.367 ± 0.239	0.837 ± 0.227	0.542 ± 0.096
GradientBoosting	0.176 ± 0.067	0.191 ± 0.214	0.443 ± 0.230	0.443 ± 0.230	0.801 ± 0.218	0.641 ± 0.111

SVM	0.143 ± 0.083	0.147 ± 0.168	0.492 ± 0.356	0.492 ± 0.356	0.643 ± 0.407	0.464 ± 0.180

Model (PRE+PERI+POST)	F1 Score	Precision	Recall	Sensitivity	Specificity	ROC-AUC
LogisticRegression	0.372 ± 0.142	0.469 ± 0.312	0.458 ± 0.222	0.458 ± 0.222	0.956 ± 0.058	0.813 ± 0.075
XGBoost	0.340 ± 0.100	0.299 ± 0.186	0.580 ± 0.247	0.580 ± 0.247	0.936 ± 0.047	0.861 ± 0.062
GradientBoosting	0.308 ± 0.089	0.383 ± 0.331	0.498 ± 0.237	0.498 ± 0.237	0.931 ± 0.070	0.839 ± 0.070
RandomForest	0.302 ± 0.081	0.340 ± 0.244	0.452 ± 0.242	0.452 ± 0.242	0.944 ± 0.049	0.782 ± 0.112
SVM	0.277 ± 0.123	0.289 ± 0.208	0.476 ± 0.262	0.476 ± 0.262	0.841 ± 0.298	0.615 ± 0.195
DecisionTree	0.232 ± 0.088	0.243 ± 0.212	0.452 ± 0.269	0.452 ± 0.269	0.876 ± 0.202	0.702 ± 0.123

Mobilisation POD2

Model (PRE)	F1 Score	Precision	Recall	Sensitivity	Specificity	ROC-AUC
LogisticRegression	0.785 ± 0.009	0.651 ± 0.015	0.989 ± 0.015	0.989 ± 0.015	0.081 ± 0.068	0.505 ± 0.057
SVM	0.781 ± 0.007	0.648 ± 0.018	0.985 ± 0.025	0.985 ± 0.025	0.072 ± 0.089	0.530 ± 0.080
GradientBoosting	0.780 ± 0.007	0.646 ± 0.015	0.985 ± 0.021	0.985 ± 0.021	0.067 ± 0.075	0.553 ± 0.046
RandomForest	0.779 ± 0.006	0.641 ± 0.013	0.995 ± 0.017	0.995 ± 0.017	0.035 ± 0.062	0.591 ± 0.050
XGBoost	0.778 ± 0.007	0.640 ± 0.013	0.994 ± 0.014	0.994 ± 0.014	0.032 ± 0.062	0.556 ± 0.053
DecisionTree	0.776 ± 0.004	0.635 ± 0.008	0.999 ± 0.006	0.999 ± 0.006	0.007 ± 0.037	0.524 ± 0.057

Model (PRE+PERI)	F1 Score	Precision	Recall	Sensitivity	Specificity	ROC-AUC
LogisticRegression	0.797 ± 0.010	0.673 ± 0.023	0.979 ± 0.027	0.979 ± 0.027	0.172 ± 0.100	0.613 ± 0.058
SVM	0.792 ± 0.012	0.667 ± 0.022	0.975 ± 0.030	0.975 ± 0.030	0.156 ± 0.099	0.618 ± 0.047
GradientBoosting	0.790 ± 0.011	0.663 ± 0.023	0.980 ± 0.026	0.980 ± 0.026	0.133 ± 0.102	0.598 ± 0.038

XGBoost	0.787 ± 0.010	0.661 ± 0.027	0.976 ± 0.034	0.976 ± 0.034	0.128 ± 0.122	0.605 ± 0.044
RandomForest	0.786 ± 0.010	0.651 ± 0.017	0.991 ± 0.017	0.991 ± 0.017	0.082 ± 0.077	0.610 ± 0.055
DecisionTree	0.779 ± 0.006	0.646 ± 0.018	0.984 ± 0.027	0.984 ± 0.027	0.064 ± 0.095	0.558 ± 0.050

**Model (PRE+PERI+POST)**	**F1 Score**	**Precision**	**Recall**	**Sensitivity**	**Specificity**	**ROC-AUC**
LogisticRegression	0.821 ± 0.022	0.711 ± 0.051	0.977 ± 0.041	0.977 ± 0.041	0.201 ± 0.196	0.639 ± 0.076
SVM	0.813 ± 0.014	0.696 ± 0.037	0.982 ± 0.040	0.982 ± 0.040	0.143 ± 0.149	0.609 ± 0.087
RandomForest	0.812 ± 0.015	0.695 ± 0.043	0.982 ± 0.033	0.982 ± 0.033	0.137 ± 0.161	0.635 ± 0.080
XGBoost	0.812 ± 0.013	0.698 ± 0.040	0.976 ± 0.047	0.976 ± 0.047	0.153 ± 0.170	0.624 ± 0.071
GradientBoosting	0.808 ± 0.014	0.688 ± 0.032	0.981 ± 0.036	0.981 ± 0.036	0.114 ± 0.141	0.624 ± 0.068
DecisionTree	0.799 ± 0.008	0.673 ± 0.032	0.989 ± 0.037	0.989 ± 0.037	0.042 ± 0.133	0.523 ± 0.081

Final model comparison

Chosen models are built with BALANCED sampling strategy and per+peri+postoperative predictors

Major Complications

**Model (BALANCED)**	**F1 Score**	**Precision**	**Recall**	**Sensitivity**	**Specificity**	**ROC-AUC**
LogisticRegression	0.400 ± 0.118	0.358 ± 0.194	0.618 ± 0.214	0.618 ± 0.214	0.943 ± 0.066	0.891 ± 0.063
GradientBoosting	0.386 ± 0.136	0.323 ± 0.198	0.658 ± 0.185	0.658 ± 0.185	0.927 ± 0.062	0.875 ± 0.095
XGBoost	0.365 ± 0.135	0.319 ± 0.202	0.602 ± 0.222	0.602 ± 0.222	0.925 ± 0.084	0.873 ± 0.072
SVM	0.351 ± 0.104	0.317 ± 0.231	0.636 ± 0.231	0.636 ± 0.231	0.922 ± 0.067	0.873 ± 0.059
RandomForest	0.342 ± 0.117	0.319 ± 0.247	0.604 ± 0.215	0.604 ± 0.215	0.926 ± 0.058	0.840 ± 0.093
DecisionTree	0.275 ± 0.131	0.204 ± 0.123	0.616 ± 0.246	0.616 ± 0.246	0.812 ± 0.309	0.736 ± 0.150

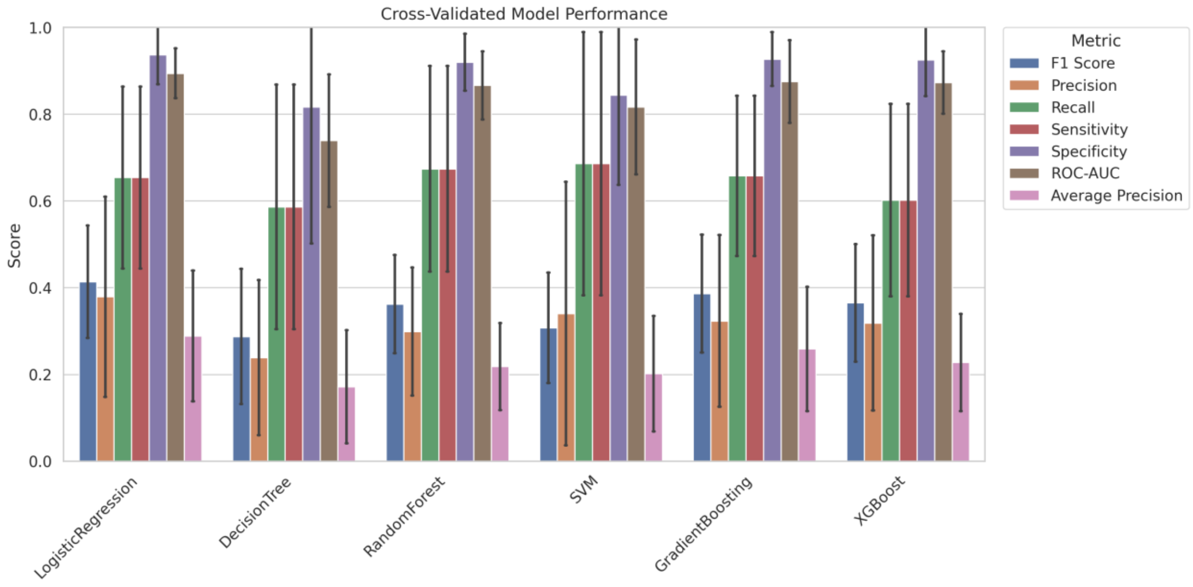

Cross-Validated Model Performance
Score
Metric
F1 Score
Precision
Recall
Sensitivity
Specificity
ROC-AUC
Average Precision
LogisticRegression
DecisionTree
RandomForest
SVM
GradientBoosting
XGBoost

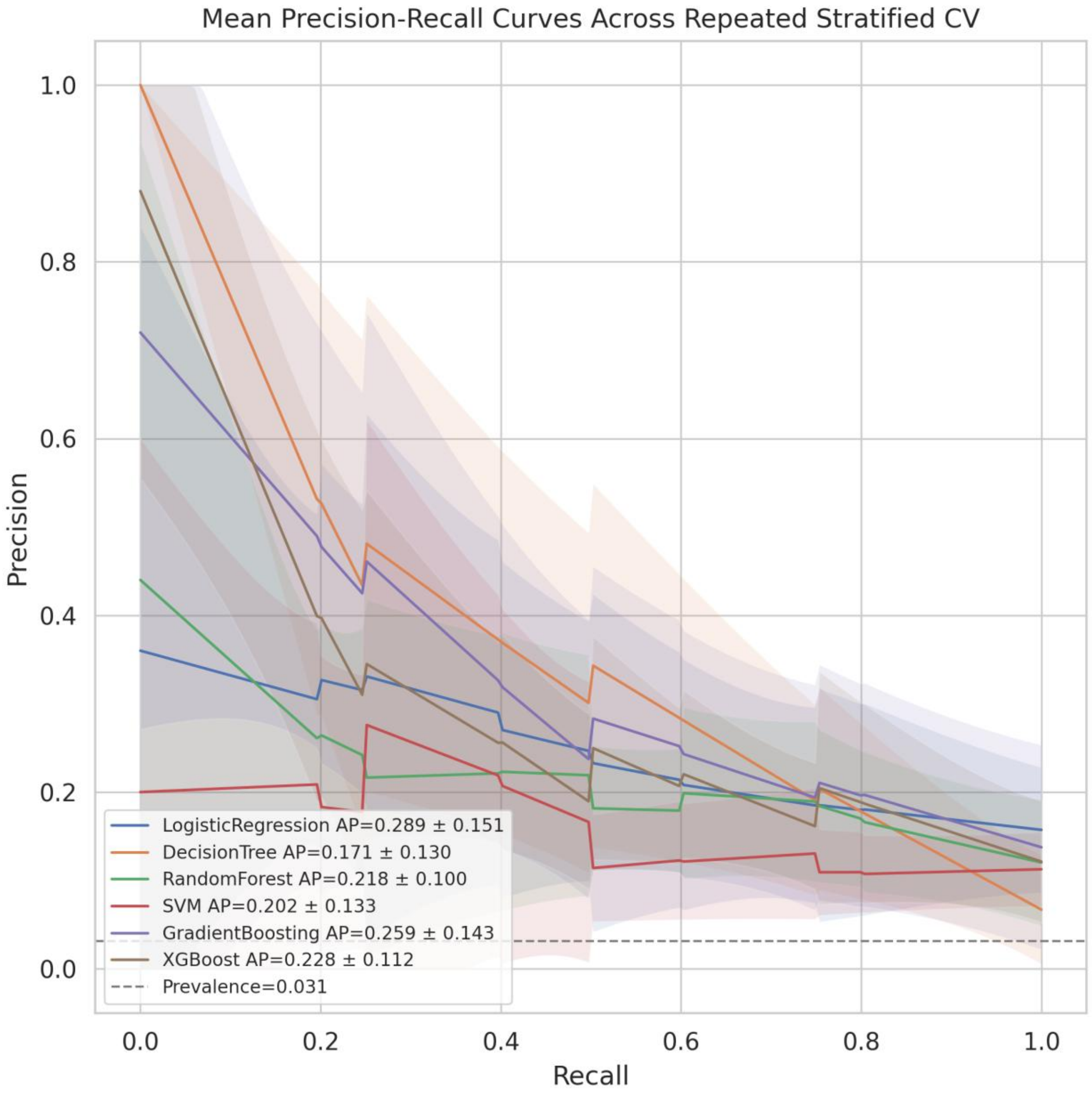

Mean Precision-Recall Curves Across Repeated Stratified CV
Precision
Recall
LogisticRegression AP=0.289 ± 0.151
DecisionTree AP=0.171 ± 0.130
RandomForest AP=0.218 ± 0.100
SVM AP=0.202 ± 0.133
GradientBoosting AP=0.259 ± 0.143
XGBoost AP=0.228 ± 0.112
Prevalence=0.031

Any Complications

Model (BALANCED)	F1 Score	Precision	Recall	Sensitivity	Specificity	ROC-AUC
LogisticRegression	0.593 ± 0.048	0.506 ± 0.080	0.743 ± 0.100	0.743 ± 0.100	0.878 ± 0.050	0.844 ± 0.043
XGBoost	0.588 ± 0.051	0.554 ± 0.098	0.650 ± 0.098	0.650 ± 0.098	0.910 ± 0.040	0.835 ± 0.038
GradientBoosting	0.572 ± 0.039	0.527 ± 0.104	0.666 ± 0.117	0.666 ± 0.117	0.895 ± 0.050	0.837 ± 0.042
RandomForest	0.564 ± 0.059	0.502 ± 0.089	0.669 ± 0.109	0.669 ± 0.109	0.890 ± 0.038	0.824 ± 0.045
SVM	0.563 ± 0.045	0.509 ± 0.127	0.699 ± 0.141	0.699 ± 0.141	0.875 ± 0.066	0.834 ± 0.037
DecisionTree	0.501 ± 0.076	0.478 ± 0.104	0.552 ± 0.105	0.552 ± 0.105	0.896 ± 0.051	0.726 ± 0.064

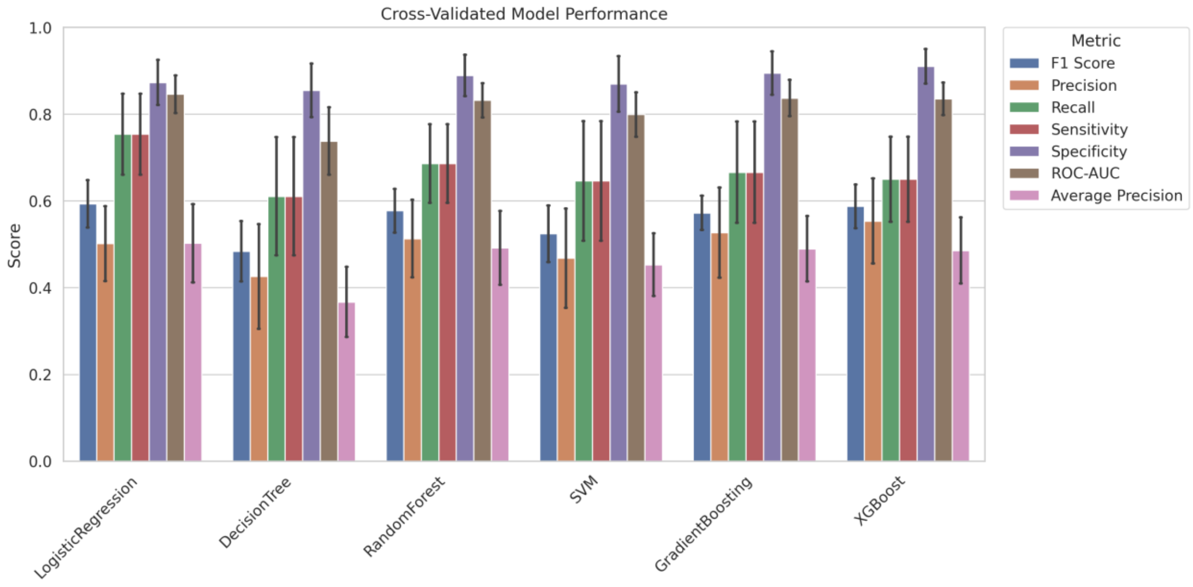
Cross-Validated Model Performance
Score
Metric
F1 Score
Precision
Recall
Sensitivity
Specificity
ROC-AUC
Average Precision
LogisticRegression
DecisionTree
RandomForest
SVM
GradientBoosting
XGBoost

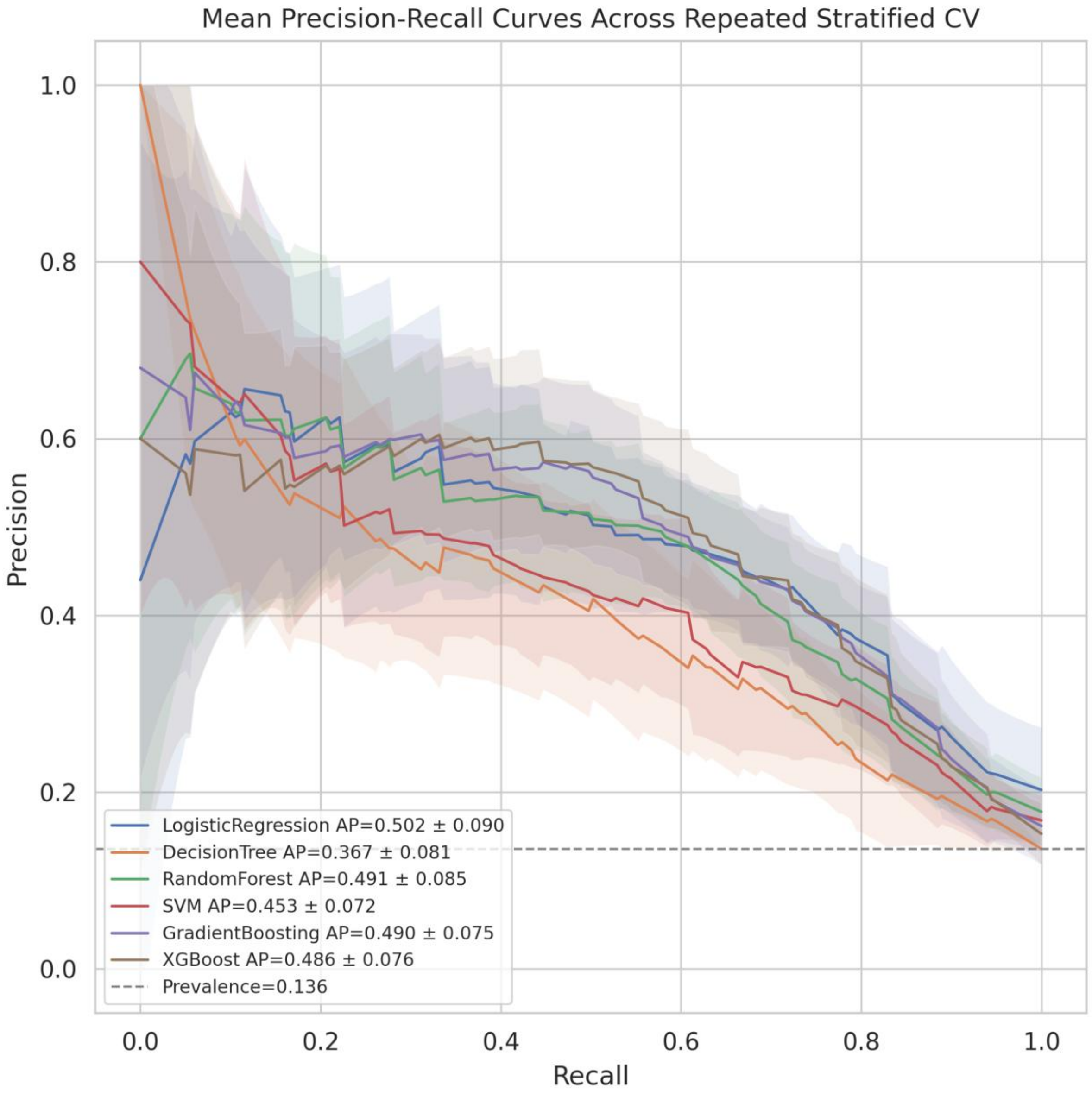
Mean Precision-Recall Curves Across Repeated Stratified CV
Precision
Recall
LogisticRegression AP=0.502 ± 0.090
DecisionTree AP=0.367 ± 0.081
RandomForest AP=0.491 ± 0.085
SVM AP=0.453 ± 0.072
GradientBoosting AP=0.490 ± 0.075
XGBoost AP=0.486 ± 0.076
Prevalence=0.136

SSI

**Model (BALANCED)**	**F1 Score**	**Precision**	**Recall**	**Sensitivity**	**Specificity**	**ROC-AUC**
LogisticRegression	0.546 ± 0.068	0.465 ± 0.112	0.697 ± 0.085	0.697 ± 0.085	0.811 ± 0.089	0.787 ± 0.051
SVM	0.535 ± 0.065	0.446 ± 0.101	0.708 ± 0.104	0.708 ± 0.104	0.795 ± 0.097	0.781 ± 0.057
XGBoost	0.513 ± 0.063	0.447 ± 0.079	0.641 ± 0.136	0.641 ± 0.136	0.819 ± 0.080	0.763 ± 0.051
GradientBoosting	0.498 ± 0.067	0.419 ± 0.096	0.667 ± 0.135	0.667 ± 0.135	0.774 ± 0.151	0.758 ± 0.053
RandomForest	0.472 ± 0.053	0.407 ± 0.123	0.635 ± 0.140	0.635 ± 0.140	0.767 ± 0.142	0.739 ± 0.052
DecisionTree	0.425 ± 0.068	0.352 ± 0.102	0.600 ± 0.150	0.600 ± 0.150	0.725 ± 0.189	0.668 ± 0.069

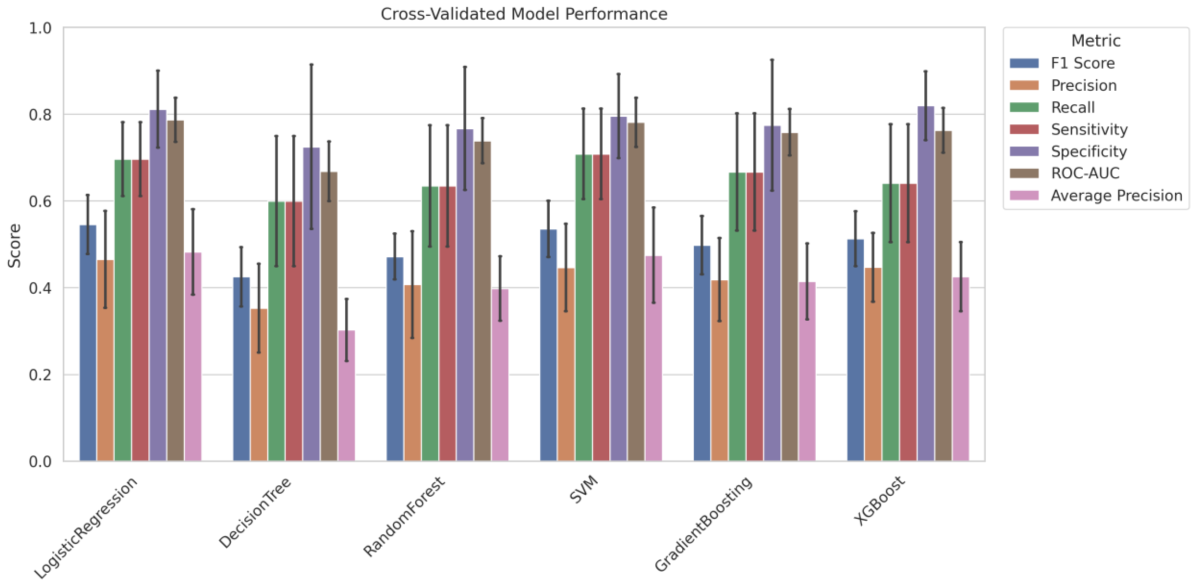
Cross-Validated Model Performance
Score
Metric
F1 Score
Precision
Recall
Sensitivity
Specificity
ROC-AUC
Average Precision
LogisticRegression
DecisionTree
RandomForest
SVM
GradientBoosting
XGBoost

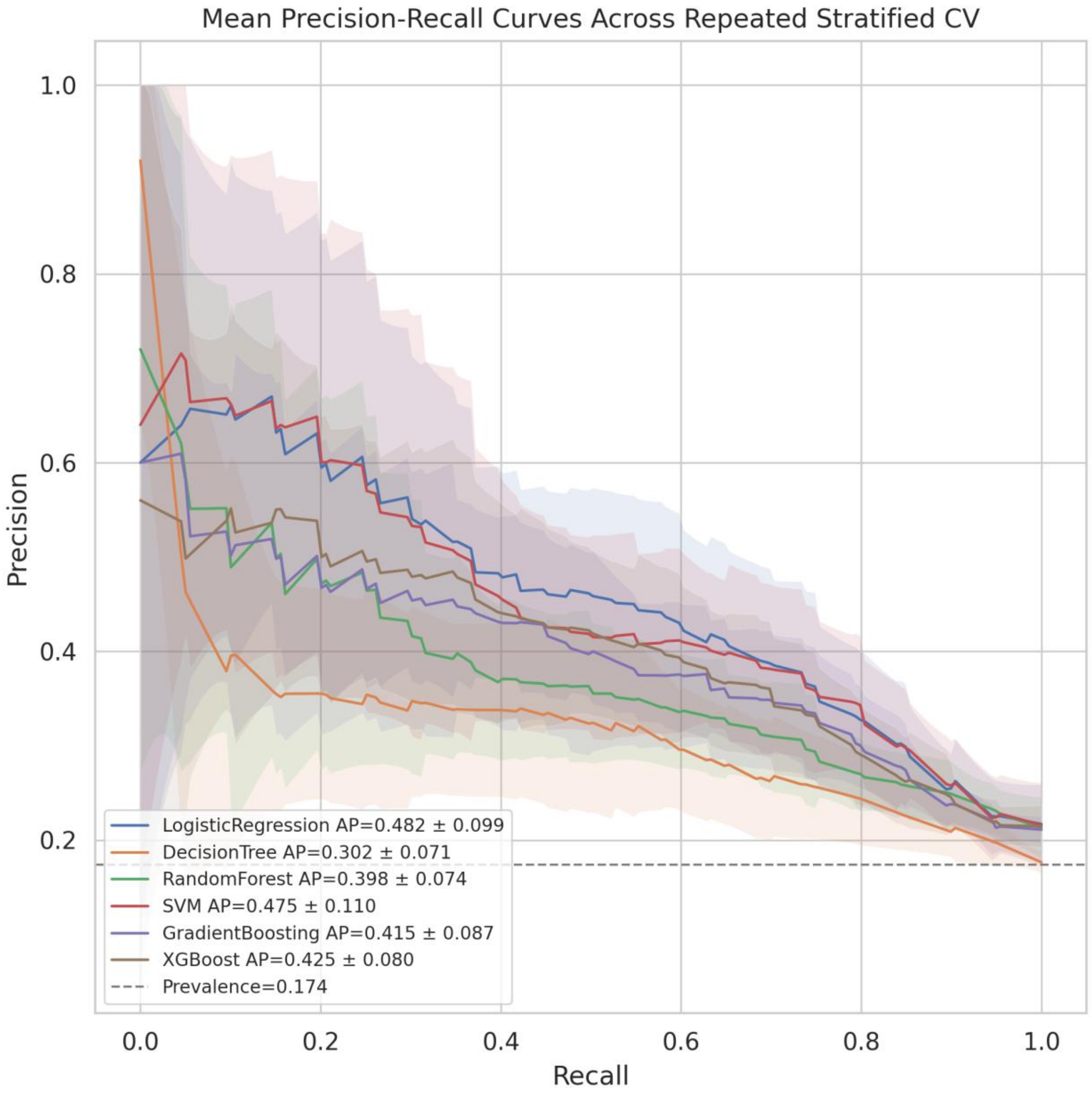
Mean Precision-Recall Curves Across Repeated Stratified CV
Precision
Recall
LogisticRegression AP=0.482 ± 0.099
DecisionTree AP=0.302 ± 0.071
RandomForest AP=0.398 ± 0.074
SVM AP=0.475 ± 0.110
GradientBoosting AP=0.415 ± 0.087
XGBoost AP=0.425 ± 0.080
Prevalence=0.174

Respiratory Complications

Model (BALANCED)	F1 Score	Precision	Recall	Sensitivity	Specificity	ROC-AUC
LogisticRegression	0.441 ± 0.125	0.440 ± 0.203	0.513 ± 0.164	0.513 ± 0.164	0.910 ± 0.082	0.743 ± 0.084
SVM	0.419 ± 0.106	0.370 ± 0.149	0.590 ± 0.156	0.590 ± 0.156	0.859 ± 0.116	0.767 ± 0.078
RandomForest	0.377 ± 0.066	0.336 ± 0.148	0.573 ± 0.203	0.573 ± 0.203	0.845 ± 0.112	0.754 ± 0.052
XGBoost	0.370 ± 0.083	0.300 ± 0.110	0.613 ± 0.203	0.613 ± 0.203	0.816 ± 0.137	0.751 ± 0.069
GradientBoosting	0.369 ± 0.074	0.273 ± 0.083	0.643 ± 0.151	0.643 ± 0.151	0.810 ± 0.087	0.756 ± 0.066
DecisionTree	0.278 ± 0.063	0.234 ± 0.093	0.473 ± 0.229	0.473 ± 0.229	0.792 ± 0.205	0.620 ± 0.089

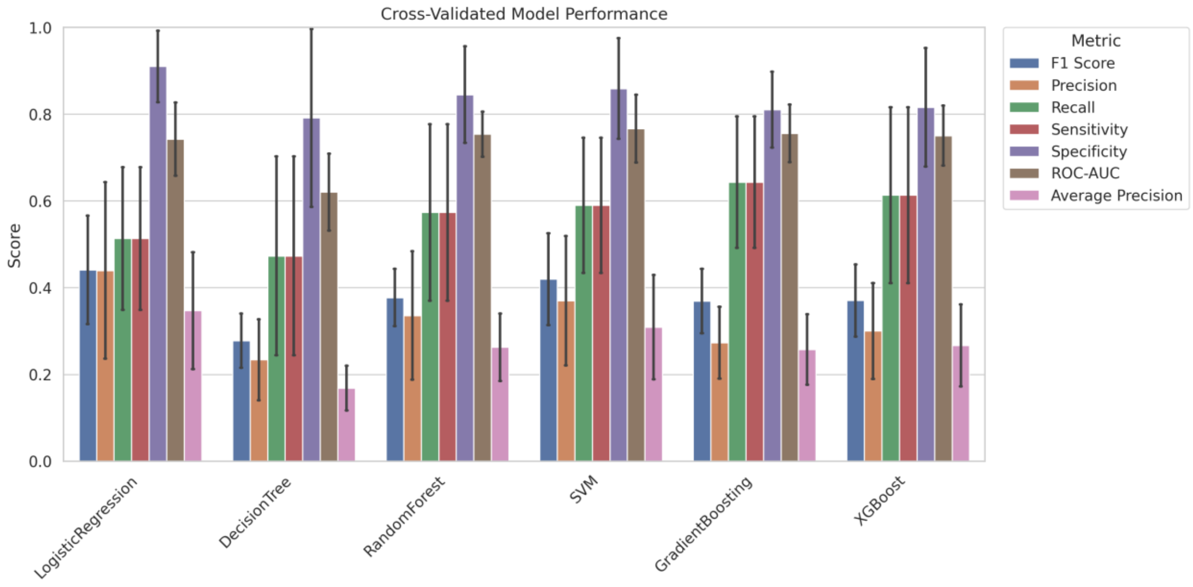

Cross-Validated Model Performance
Score
Metric
F1 Score
Precision
Recall
Sensitivity
Specificity
ROC-AUC
Average Precision
LogisticRegression
DecisionTree
RandomForest
SVM
GradientBoosting
XGBoost

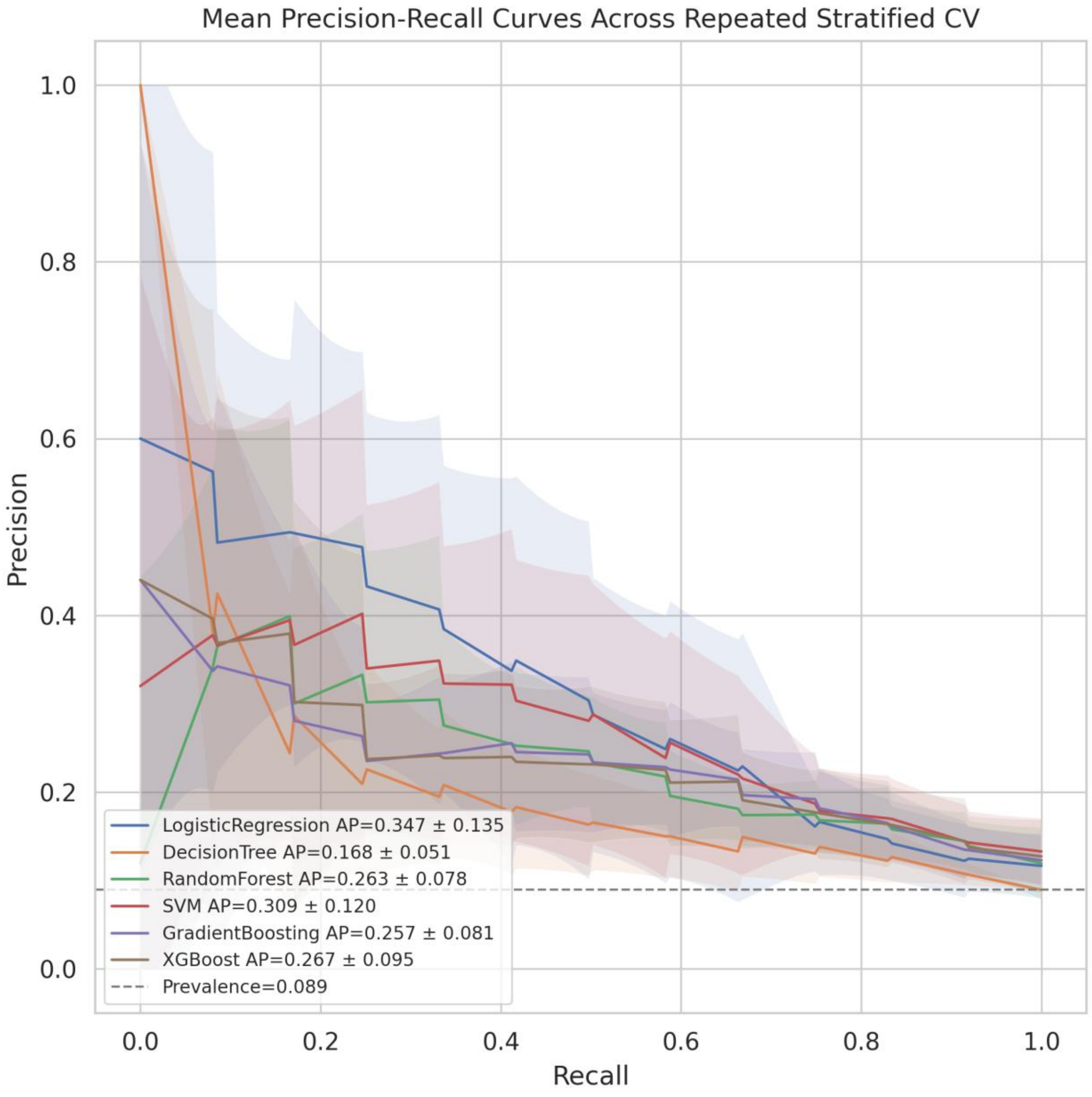

Mean Precision-Recall Curves Across Repeated Stratified CV
Precision
Recall
LogisticRegression AP=0.347 ± 0.135
DecisionTree AP=0.168 ± 0.051
RandomForest AP=0.263 ± 0.078
SVM AP=0.309 ± 0.120
GradientBoosting AP=0.257 ± 0.081
XGBoost AP=0.267 ± 0.095
Prevalence=0.089

POI

Model (BALANCED)	F1 Score	Precision	Recall	Sensitivity	Specificity	ROC-AUC
SVM	0.626 ± 0.116	0.677 ± 0.180	0.627 ± 0.174	0.627 ± 0.174	0.921 ± 0.059	0.782 ± 0.100
LogisticRegression	0.620 ± 0.125	0.617 ± 0.184	0.691 ± 0.172	0.691 ± 0.172	0.867 ± 0.172	0.776 ± 0.095
GradientBoosting	0.612 ± 0.144	0.693 ± 0.228	0.611 ± 0.166	0.611 ± 0.166	0.896 ± 0.167	0.743 ± 0.085
XGBoost	0.586 ± 0.143	0.704 ± 0.249	0.594 ± 0.200	0.594 ± 0.200	0.885 ± 0.181	0.749 ± 0.087
RandomForest	0.585 ± 0.121	0.656 ± 0.231	0.594 ± 0.152	0.594 ± 0.152	0.899 ± 0.097	0.775 ± 0.094
DecisionTree	0.459 ± 0.125	0.506 ± 0.234	0.581 ± 0.248	0.581 ± 0.248	0.726 ± 0.376	0.636 ± 0.102

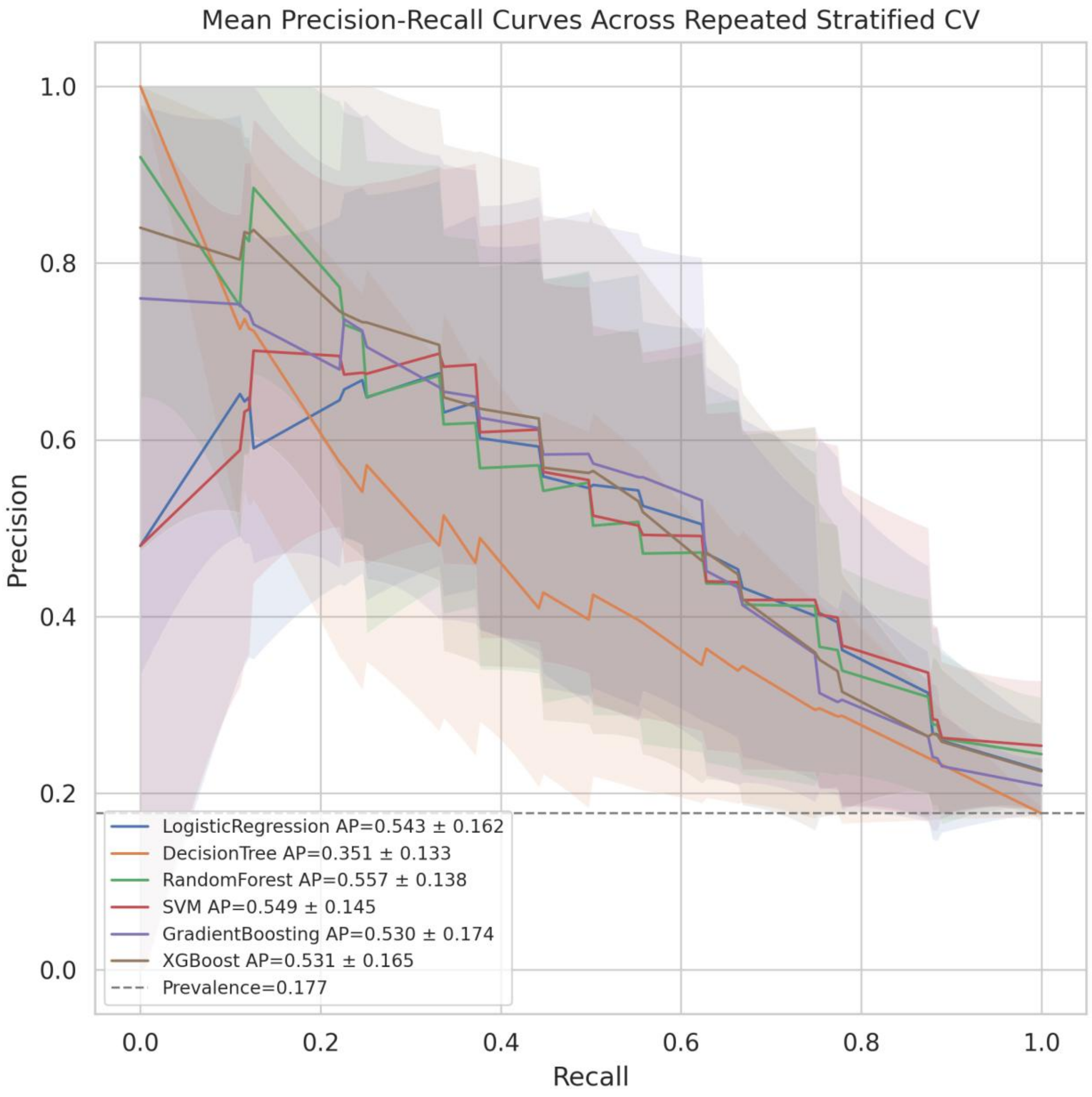

Renal Dysfunction

Model (BALANCED)	F1 Score	Precision	Recall	Sensitivity	Specificity	ROC-AUC
LogisticRegression	0.381 ± 0.152	0.459 ± 0.319	0.488 ± 0.220	0.488 ± 0.220	0.948 ± 0.077	0.807 ± 0.075
XGBoost	0.340 ± 0.100	0.299 ± 0.186	0.580 ± 0.247	0.580 ± 0.247	0.936 ± 0.047	0.861 ± 0.062
RandomForest	0.320 ± 0.113	0.389 ± 0.324	0.472 ± 0.258	0.472 ± 0.258	0.948 ± 0.045	0.793 ± 0.108
GradientBoosting	0.308 ± 0.089	0.383 ± 0.331	0.498 ± 0.237	0.498 ± 0.237	0.931 ± 0.070	0.839 ± 0.070

SVM	0.285 ± 0.075	0.274 ± 0.243	0.626 ± 0.285	0.626 ± 0.285	0.896 ± 0.080	0.828 ± 0.073
DecisionTree	0.186 ± 0.122	0.229 ± 0.277	0.508 ± 0.350	0.508 ± 0.350	0.650 ± 0.456	0.573 ± 0.100

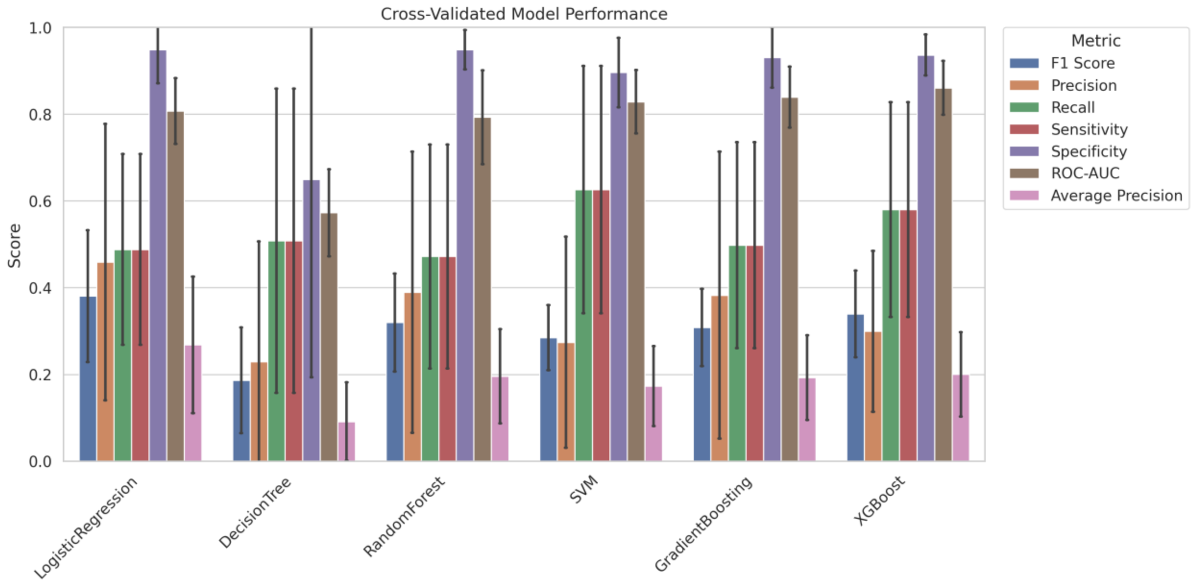
Cross-Validated Model Performance
Score
Metric
F1 Score
Precision
Recall
Sensitivity
Specificity
ROC-AUC
Average Precision
LogisticRegression
DecisionTree
RandomForest
SVM
GradientBoosting
XGBoost

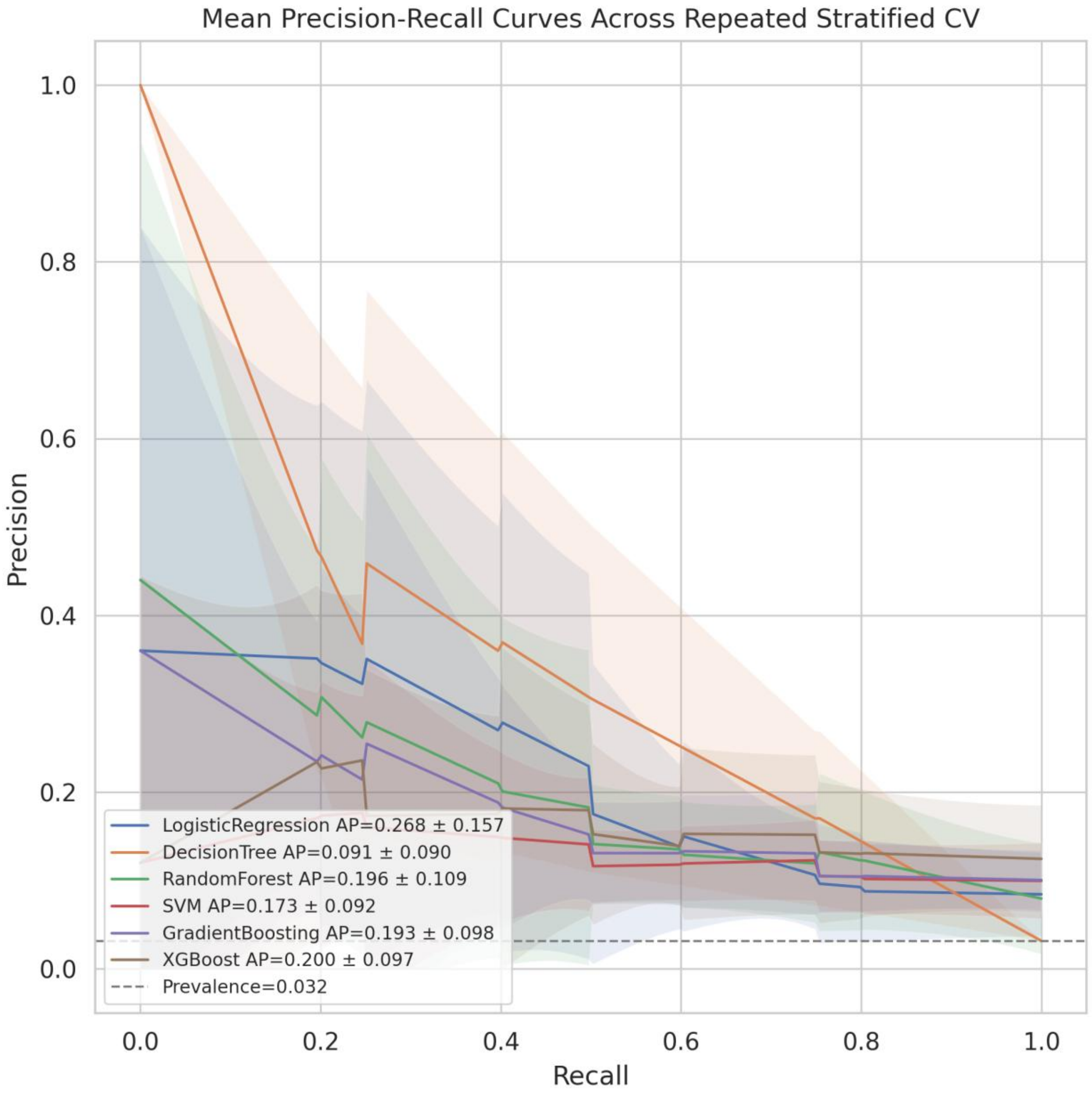
Mean Precision-Recall Curves Across Repeated Stratified CV
Precision
Recall
LogisticRegression AP=0.268 ± 0.157
DecisionTree AP=0.091 ± 0.090
RandomForest AP=0.196 ± 0.109
SVM AP=0.173 ± 0.092
GradientBoosting AP=0.193 ± 0.098
XGBoost AP=0.200 ± 0.097
Prevalence=0.032

Mobilisation on POD2

Model (BALANCED)	F1 Score	Precision	Recall	Sensitivity	Specificity	ROC-AUC
LogisticRegression	0.820 ± 0.022	0.709 ± 0.052	0.978 ± 0.039	0.978 ± 0.039	0.192 ± 0.198	0.638 ± 0.078
SVM	0.812 ± 0.012	0.702 ± 0.046	0.970 ± 0.051	0.970 ± 0.051	0.173 ± 0.174	0.623 ± 0.113
XGBoost	0.812 ± 0.013	0.698 ± 0.040	0.976 ± 0.047	0.976 ± 0.047	0.153 ± 0.170	0.624 ± 0.071
RandomForest	0.811 ± 0.018	0.695 ± 0.042	0.978 ± 0.036	0.978 ± 0.036	0.143 ± 0.167	0.615 ± 0.074
GradientBoosting	0.808 ± 0.014	0.688 ± 0.032	0.981 ± 0.036	0.981 ± 0.036	0.114 ± 0.141	0.624 ± 0.068
DecisionTree	0.800 ± 0.009	0.669 ± 0.019	0.996 ± 0.013	0.996 ± 0.013	0.027 ± 0.076	0.547 ± 0.067

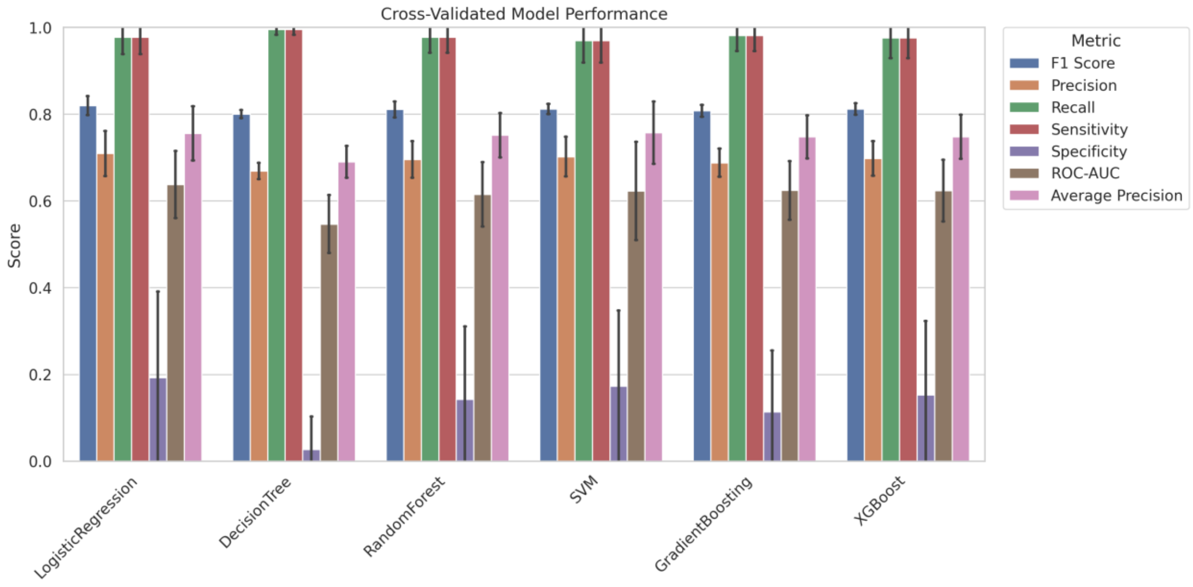
Cross-Validated Model Performance
Score
Metric
F1 Score
Precision
Recall
Sensitivity
Specificity
ROC-AUC
Average Precision
LogisticRegression
DecisionTree
RandomForest
SVM
GradientBoosting
XGBoost

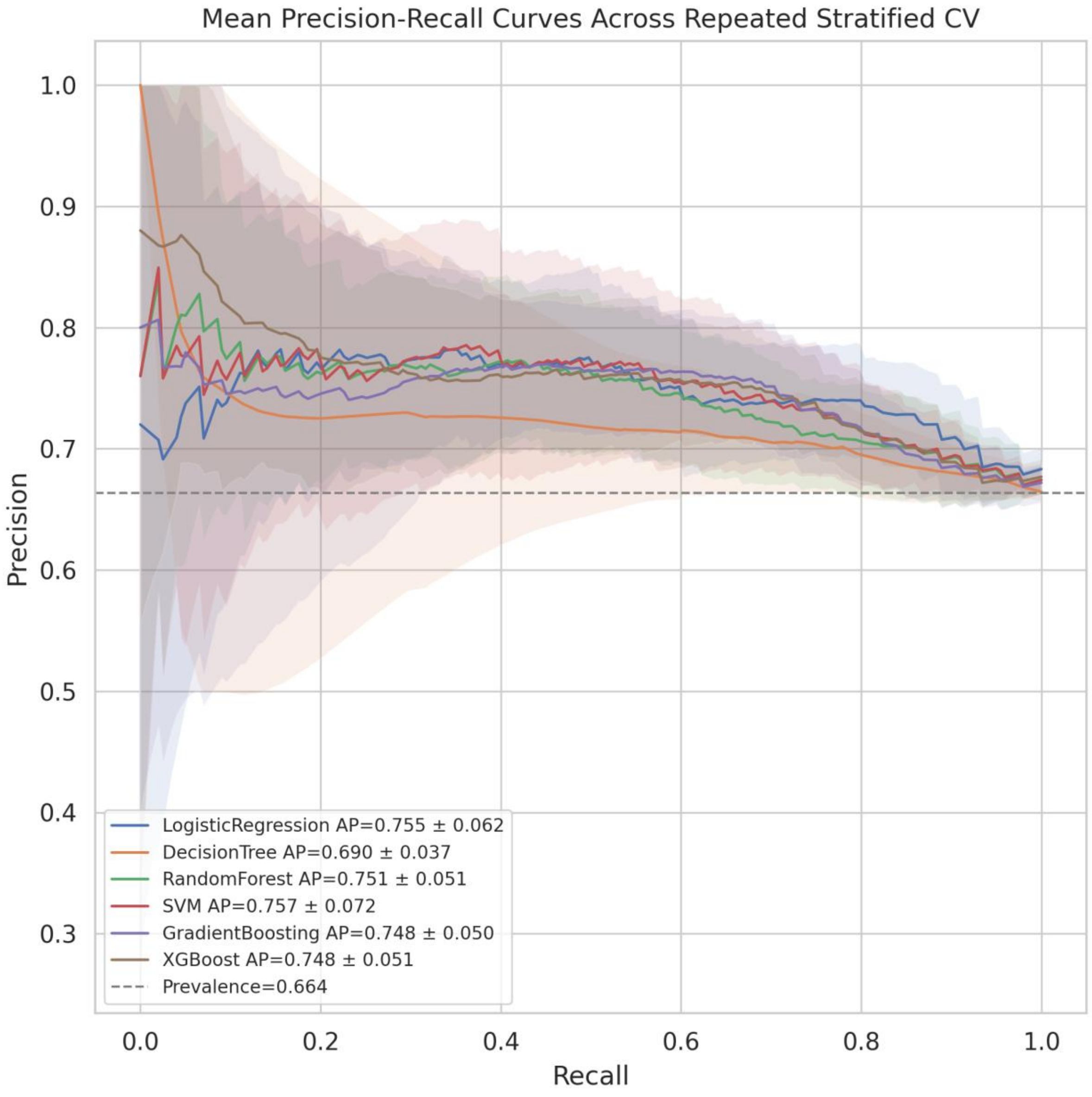
Mean Precision-Recall Curves Across Repeated Stratified CV
Precision
Recall
LogisticRegression AP=0.755 ± 0.062
DecisionTree AP=0.690 ± 0.037
RandomForest AP=0.751 ± 0.051
SVM AP=0.757 ± 0.072
GradientBoosting AP=0.748 ± 0.050
XGBoost AP=0.748 ± 0.051
Prevalence=0.664

SHAP feature rankings for all variables

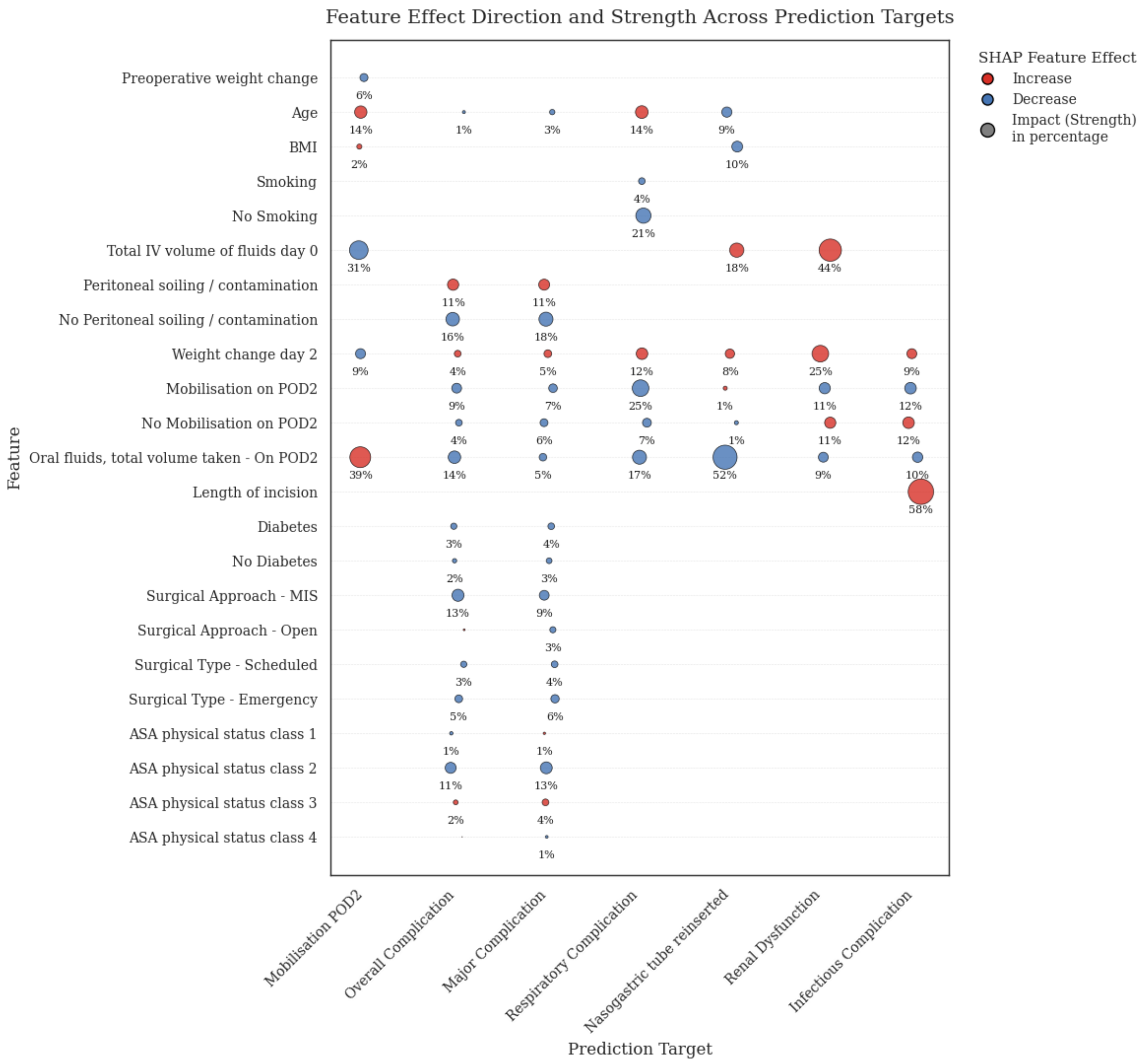

Figure. Summary of SHAP feature importance across multiple prediction targets. Bubble size represents the relative impact strength (percentage contribution) of each feature, while color indicates the direction of effect on the model prediction (red: increase, blue: decrease). The figure highlights both shared and outcome-specific predictors, illustrating how clinical features differentially influence any, major, respiratory and infectious complications, delayed mobilization, and renal dysfunction. BMI – body mass index, POD – postoperative day
